# Label-free classification of cells based on supervised machine learning of subcellular structures

**DOI:** 10.1371/journal.pone.0211347

**Published:** 2019-01-29

**Authors:** Yusuke Ozaki, Hidenao Yamada, Hirotoshi Kikuchi, Amane Hirotsu, Tomohiro Murakami, Tomohiro Matsumoto, Toshiki Kawabata, Yoshihiro Hiramatsu, Kinji Kamiya, Toyohiko Yamauchi, Kentaro Goto, Yukio Ueda, Shigetoshi Okazaki, Masatoshi Kitagawa, Hiroya Takeuchi, Hiroyuki Konno

**Affiliations:** 1 Second Department of Surgery, Hamamatsu University School of Medicine, Hamamatsu, Shizuoka, Japan; 2 Central Research Laboratory, Hamamatsu Photonics K.K., Hamamatsu, Shizuoka, Japan; 3 Department of Medical Spectroscopy, Institute for Medical Photonics Research, Preeminent Medical Photonics Education and Research Center, Hamamatsu University School of Medicine, Hamamatsu, Shizuoka, Japan; 4 Department of Molecular Biology, Hamamatsu University School of Medicine, Hamamatsu, Shizuoka, Japan; 5 Laboratory Animal Facilities and Services, Preeminent Medical Photonics Education and Research Center, Hamamatsu University School of Medicine, Hamamatsu, Shizuoka, Japan; 6 Hamamatsu University School of Medicine, Hamamatsu, Shizuoka, Japan; Zapadoceska univerzita, CZECH REPUBLIC

## Abstract

It is demonstrated that cells can be classified by pattern recognition of the subcellular structure of non-stained live cells, and the pattern recognition was performed by machine learning. Human white blood cells and five types of cancer cell lines were imaged by quantitative phase microscopy, which provides morphological information without staining quantitatively in terms of optical thickness of cells. Subcellular features were then extracted from the obtained images as training data sets for the machine learning. The built classifier successfully classified WBCs from cell lines (area under ROC curve = 0.996). This label-free, non-cytotoxic cell classification based on the subcellular structure of QPM images has the potential to serve as an automated diagnosis of single cells.

## Introduction

Morphological classification of cells and tissue on a subcellular scale under a microscope has a long history in pathology, including cytology and histology. The subcellular organelles cause subcellular features such as increased nuclear-to-cytoplasmic ratio, granular cytoplasm, and a large round nucleus with prominent nucleolus [1]. In identifying and classifying diseases, physicians recognize and analyze the pattern (features) in the microscopic image and interpret its meaning from their past training (experience). In cell biology, cytology, and pathology, the features of cells or tissue to be recognized and analyzed can be enhanced in two ways: one is staining with dyes or labeling the molecules to be observed with fluorescence light; the other is optical filtering by dark- or bright-field microscopy, including label-free optical imaging such as phase-contrast and differential-interference-contrast imaging. The former describes subcellular features as a distribution map of specific proteins or molecules. The later describes the features as a refractive index map of various proteins or molecules. In this paper, we refer to the refractive index map inside a cell as a subcellular structure.

For the last decade, as one task in computer vision, pattern recognition has been the most-topical area in fields such as autonomous cars and security. In these fields, patterns on target objects are described in certain manners, such as Haar-like features, local binary patterns (LBPs), and histogram of oriented gradients (HOG) [2][3], to enhance features of imagery or suppress artifacts such as illumination because general video or still cameras offer images as they are (i.e., no staining, labelling, or optical filtering). The combination of pattern recognition and machine learning is opening up new fields in not only industry but also biomedical and medical imaging.

Recently, as artificial intelligence becomes more advanced, automated diagnosis [4] of tissue and cells is gaining popularity. In such diagnosis, the texture of labeled or stained tissue slices and cells are automatically recognized and classified as normal or abnormal by computer vision trained by machine learning. On the other hand, label-free automated detection [5–8] and classification [9–13] of single cells (not in tissue) have been developed over the last decade or so. For instance, cells have been classified via imaging-flow cytometry in a manner of bright-field microcopy except quantitative-phase microscopy (QPM), dark-field microscopy, and machine learning of subcellular morphology [14]. Also, label-free drug assessment of cells is performed in the same manner as imaging-flow cytometry [15]. These two applications of imaging-flow cytometry utilize optical filtering to enhance the subcellular features of single cells.

Among the types of bright-field microscopy, QPM complements [16] the existing conventional label-free interferometric imaging, namely, phase-contrast microscopy, from the viewpoint of giving quantitative information about optical-path length of specimens and suppressing artifacts such as the “halo” [17–20] and “shade off” that hinder image segmentation [20]. Since QPM provides a non-optically-filtered image about optical path-length (OPL), a QPM image consists of all spatial-frequency components. The drawback of QPM images is that the subcellular structure is “buried” in the exiting spatial low-frequency components; consequently, instead of subcellular structure, physical parameters (physical features such as cellular volume, surface area, sphericity, dry mass, and dry mass density) in a QPM image have been be utilized for cell classification [16,21–23]. Here we refer to the physical parameters as cellular outlines to be contrasted with subcellular structures. A set of statistics of subcellular structures (standard deviations, variances, skewness, kurtosis, and so on) of a QPM image have been also utilized for cell classification by means of machine learning [24,25].

In consideration of pathological diagnosis, namely, a doctor diagnoses dysplasia of cells through a microscope on the basis of not only such cellular outline but also the subcellular structure of the tissue slices or cells stained, the subcellular structure of a single cell should also be extracted (recognized) and on the basis of which, cells are classified when computer vision also diagnoses. Among the various algorithms for extracting patterns from an image, histograms of oriented gradients [26] (HOG) is a de-facto standard for detecting humans by computer vision. HOG is generally variant to image size and rotation. To the best of our knowledge, HOG-based feature extraction has not been applied to QPM images of label-free single-cells, although modified HOG has been used to extract features from an image of stained cells [27]. Alternative method for extracting patterns from an image and classifying them is deep-learning [28].

In this paper, we describe a shallow-learning [29] approach for classifying rule-based auto-segmented images of healthy white blood cells (WBCs) and cancer cell-lines (CLs) on the basis of the subcellular structure in QPM images. After the sizes of the cells in the QPM images were longitudinally and laterally normalized, the subcellular structure was extracted by the HOG-based feature-extraction algorithm. A support vector machine [30–32] (SVM) classifier was trained on the subcellular features by HOG descriptor, and the classification performance was plotted as a detection-error trade-off curve [33] (DET). For comparison, another SVM classifier was also trained by using a set of statistics of subcellular structures (statistical subcellular-structures).

## Materials and methods

### Quantitative phase microscopy

**Fig 1:**
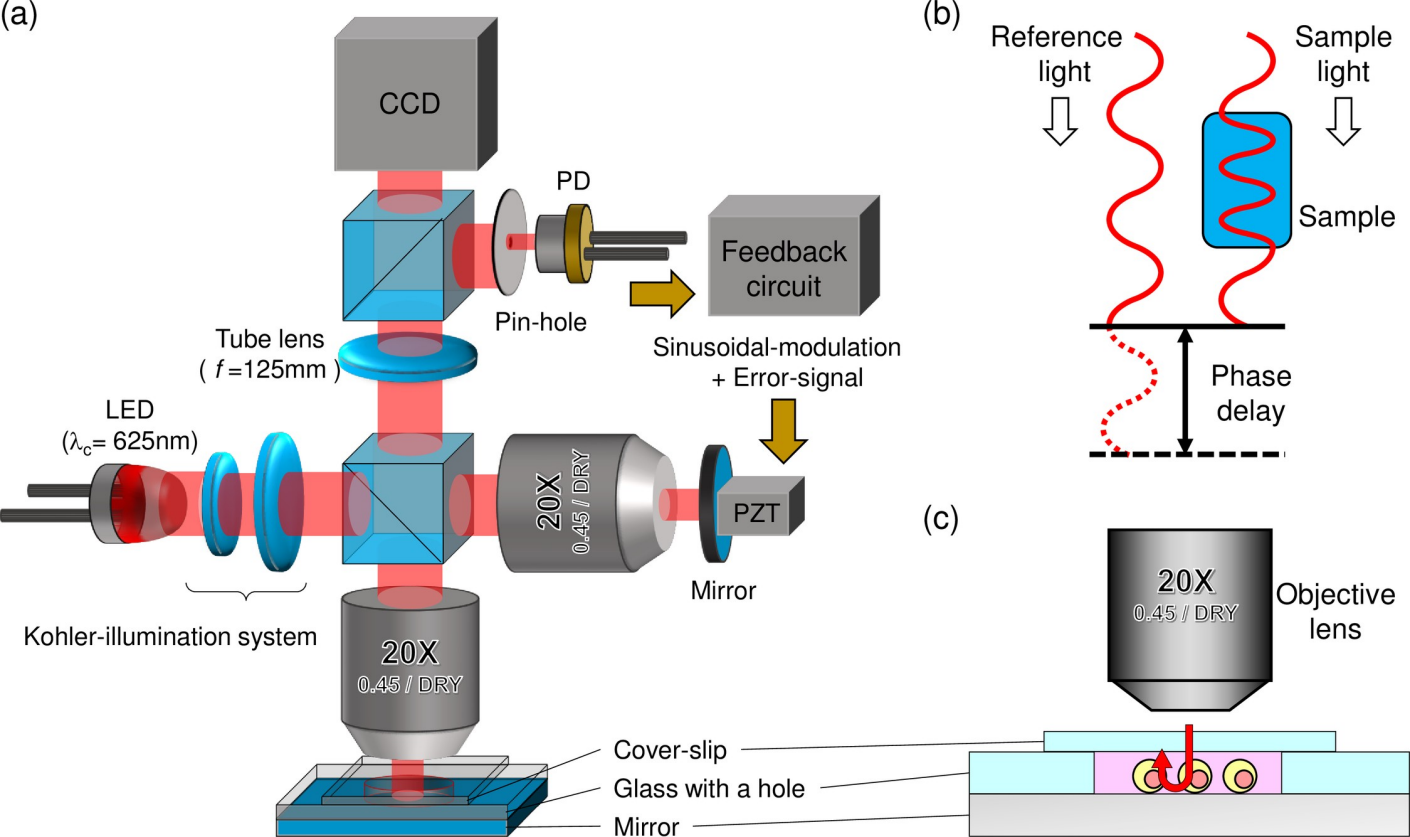
Schematic of imaging system and principle of phase measurement. (a) Schematic of reflection-type quantitative phase microscope. (b) Principle of phase measurement of the sample in QPM. (c) Configuration of custom-made slide chamber for cells in reflection-type QPM.

Among the various label-free imaging techniques, actively stabilized phase-shifting reflection-type QPM [34–37]—one kind of bright-field microcopy—was used in this study (Fig 1(A)). It provides quantitative morphological information about live cells without need for cytotoxic methods such as photo-bleaching and photo-toxicity [17], which are commonly used in fluorescence-labeled microscopy. As shown schematically in Fig 1(B), the difference between the refractive index (RI) of a cell (n_1_) and that of its surrounding medium (n_0_) and the physical path length (PL) cause phase delay of the incident light (wavelength; λ) to a sample according to Eq (1).

$$\Delta \phi (x,y)=\frac{2\pi }{\lambda }\left(n_{1}-n_{0}\right)\cdot 2PL(x,y)=\frac{4\pi }{\lambda }OPL(x,y)$$ (1)

The phase delay (Δϕ) of the incident light is referred as the “optical path length” (OPL) of an object. RI acts as an intrinsic contrast agent [38] that enhances contrast of transparent samples. In the reflection-type configuration of a QPM shown in Fig 1(C), light incident on the sample passes through the sample twice; therefore, according to Eq (1), the physical path length (PL) is doubled.

In our QPM shown in Fig 1(A), the imaging light emitted from an LED (center wavelength: 625 nm) is split into two lights: one is incident on the sample, and the other is modulated (in phase) by a mirror mounted on the PZT transducer. These lights generate an interference pattern called “interferograms.” The phase delay is retrieved from the interferograms by applying a temporal-phase-shifting algorithm. Magnification of our QPM is 13.89×, and the numerical aperture of the objective lens (Olympus 20×) is 0.45. The theoretical depth of focus is 1.5 μm. Tube lens with focal length of 125 mm is used for the objective lens (designed for a tube length of 180 mm). Interferograms are acquired by a two-dimension-arrayed sensor (CCD) with a pixel size of 3.75 μm and 12-bit depth (Guppy Pro F-125B, Allied Vision Technologies GmbH). The histogram of the background noise in a phase image was shown in S1 Fig. The standard deviation of the background noise was about 5 nm (50 milli-radians). The cells in the phosphate buffered-saline medium are configured in a custom-made slide chamber, as shown in Fig 1(C), where the slide underneath the cells is reflection coated to make the light passing through the cells reflect back to the objective lens. The reflection-coated glass slide is covered by a coverslip with a gap of around 1 mm so that the cells are not compressed and maintain their shape.

### Rule-based auto-segmentation of cells

**Fig 2:**
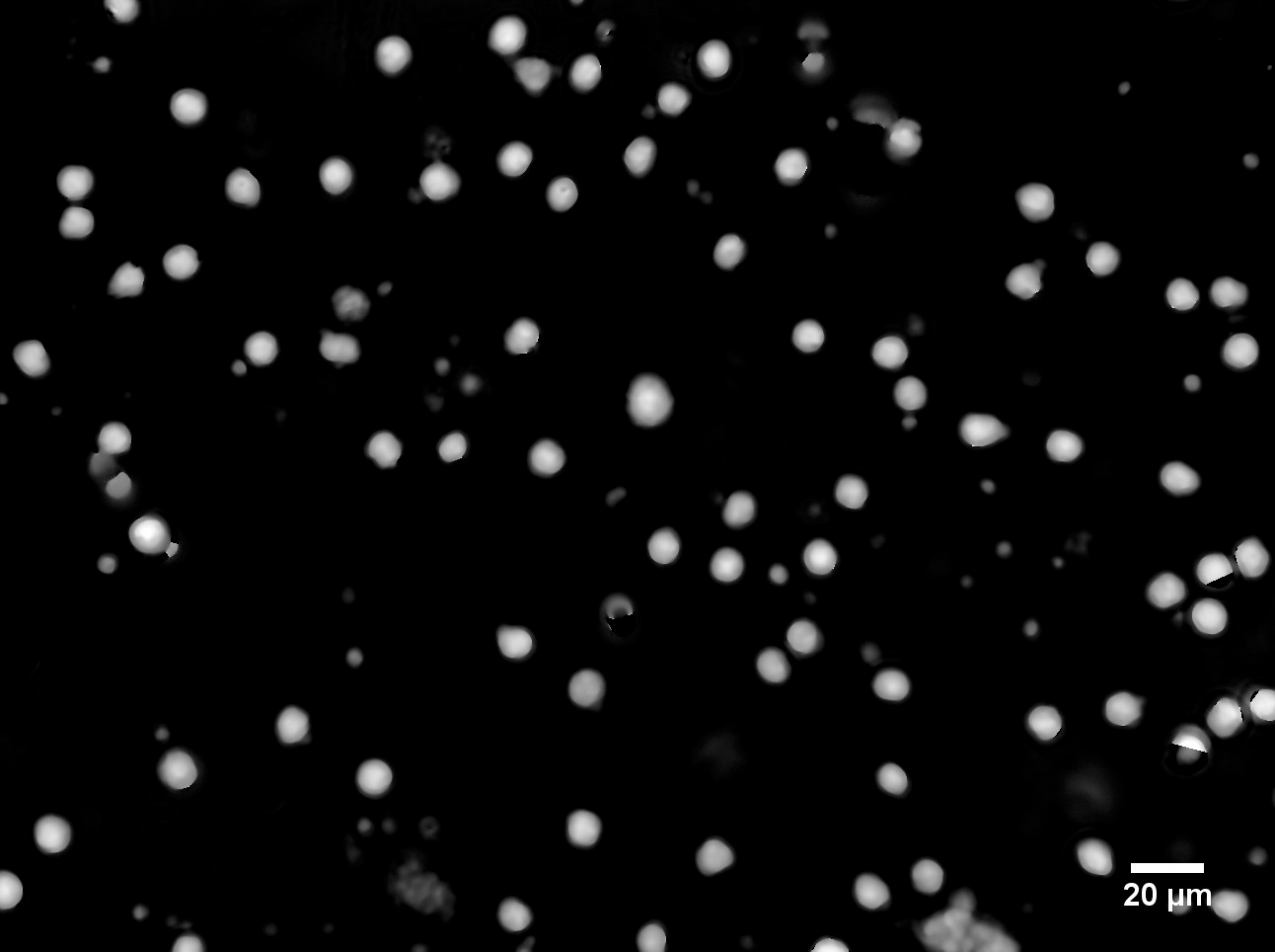
Full-field of view of a QPM image of WBCs after the removal of curvature in phase. White spots are QPM images of single WBCs, and the black region is background. Image size is 350×260 μm (1292×964 pixels). In the sample preparation, only WBCs were extracted from whole blood and then suspended in PBS.

**Fig 3:**
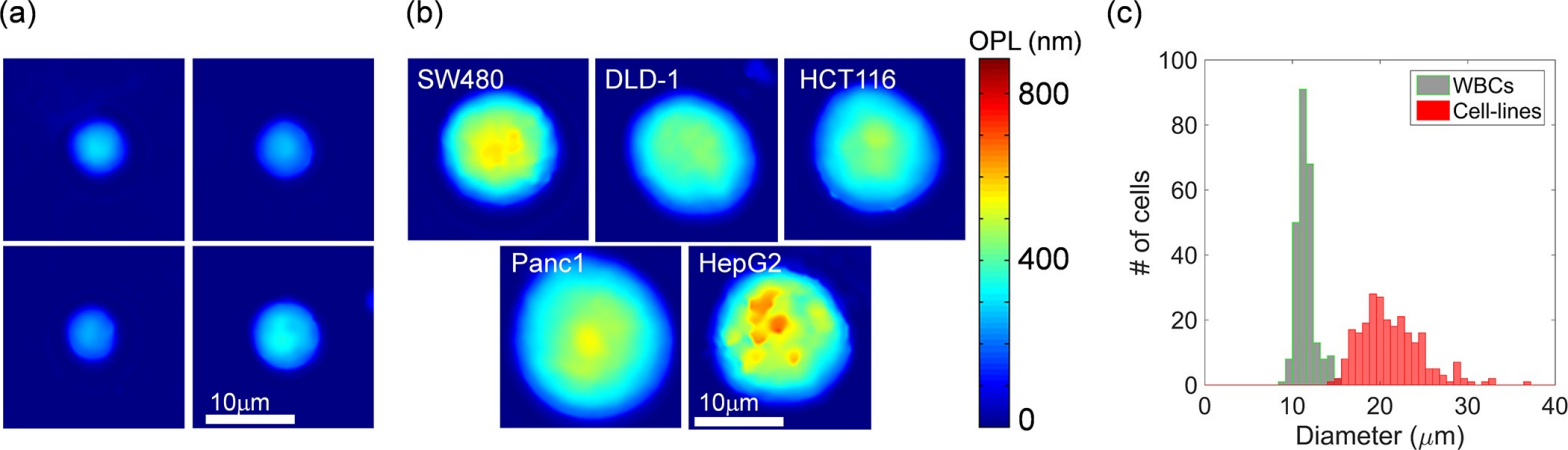
Representative segmented QPM images of single cells and their size distribution. (a) QPM images of WBC of a healthy donor. (b) QPM images of five types of cancer cell lines, namely, SW480, DLD-1, HCT116, Panc-1, and HepG2. Images were captured immediately after trypsinization to imitate CTCs floating in circulation. Pseudo-color represents OPL in nanometers. (c) Size distributions of diameters of 250 WBCs (green bars) and 250 cell lines (red bars).

Our QPM provides a field of view of 350×260 μm (Fig 2). The background of the acquired QPM image typically has curvature in terms of phase because of the mismatch of the curvatures of the wavefronts of the sample- and reference lights. After the curvature is removed (the procedure was shown in S2 Fig), the backgrounds of the image are uniform, so it is easy to segment each cell on the basis of a triangle algorithm [39], which is one of the auto thresholding algorithms implemented in the open-source image-processing software ImageJ. Representative segmented QPM images of WBCs of a healthy donor and five types of cancer cell lines (SW480, DLD-1, HCT116, Panc-1, and HepG2) are shown in pseudo color in Fig 3(A) and 3(B), respectively. Since the difference between the diameters of the WBCs and the CLs is obvious in Fig 3(C), and it is easy to classify them, the sizes of cells were normalized to emphasize the classification based on subcellular structure by the method described in following section.

### OPL normalizations for size-invariant feature extraction

**Fig 4:**
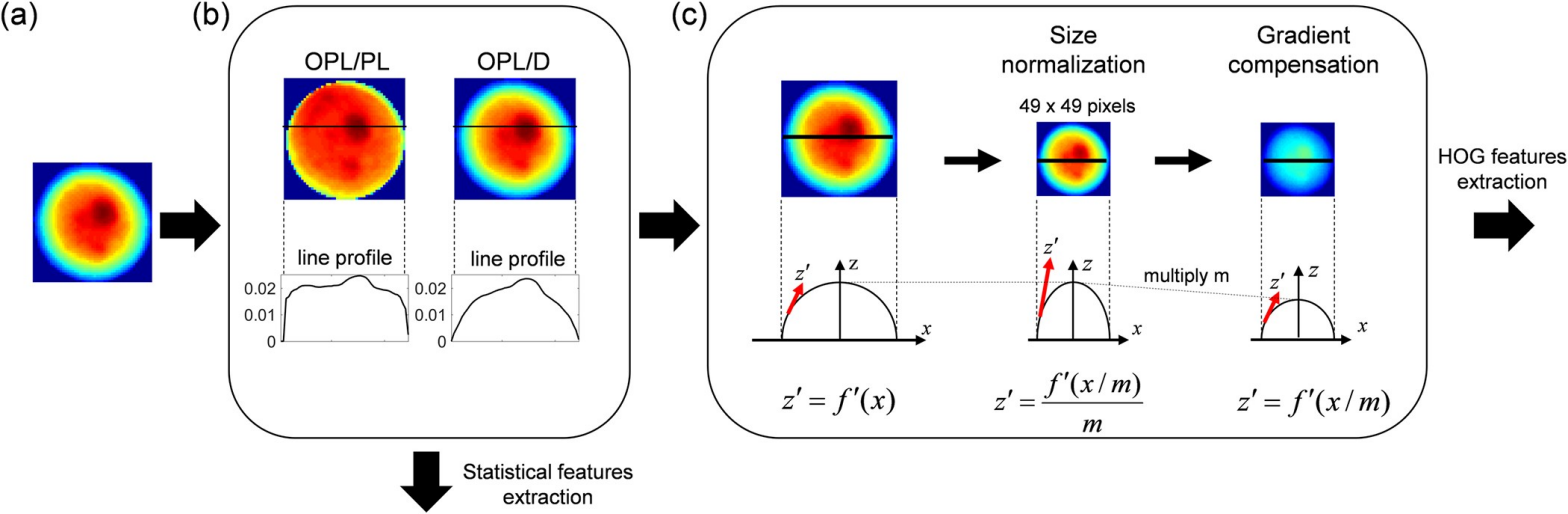
Two-branch flowchart of preprocessing for size-invariant feature extraction for statistical- and HOG-based subcellular classification. (a) Segmented image of a cell. (b) OPL normalizations by path length (PL) or diameter (D). Line profiles along the black lines in the pseudo color images of OPL/PL or OPL/D are shown for comparison of two normalizations. The details of subcellular structure are unclear in the profile of OPL/D because of spatial low frequency components (the hemisphere), whereas the details of intracellular structure are recognized in the flat region of the profile of OPL/PL. (c) Size normalization and gradient compensation. Function *f* represents the profile of OPL/D or OPL/PL. Red arrows represent the spatial gradients of *f* (i.e., *f*′). The gradients were divided by resize factor *m* for compensations.

To eliminate the influence of size on the classification, longitudinal (OPL) and lateral (size) directions of QPM images should be considered, as shown in Fig 4. Statistical features such as mean and standard deviation are already normalized so they are not influenced by the size of cells. In the longitudinal direction, as the size of cells increases, OPLs in the QPM images get thicker, because according to Eq (1), the OPLs are proportional to the thickness and RI. One longitudinal normalization is done by dividing OPLs by diameter (D) as representative thickness. The diameter can be predicted from a two-dimensional image under the assumption that that a floating cell is a sphere. Another longitudinal normalization is also proposed hereafter. Under the same assumption that a floating cell is almost a sphere with radius D/2, its path length along which light penetrates can be expressed as follows:

$$PL(x,y)=2\sqrt{{\left(D/2\right)}^{2}-x^{2}-y^{2}}.$$ (2)

OPLs are divided by path length (PL) as real (not representative) thickness. A QPM image divided by its diameter or path length and line profiles along the solid lines in the images are shown in Fig 4(B). The ratio of OPL to PL or the ratio of OPL to D is a representative parameter as a dimensionless number.

### Statistical subcellular-structures of single cells

**Fig 5:**
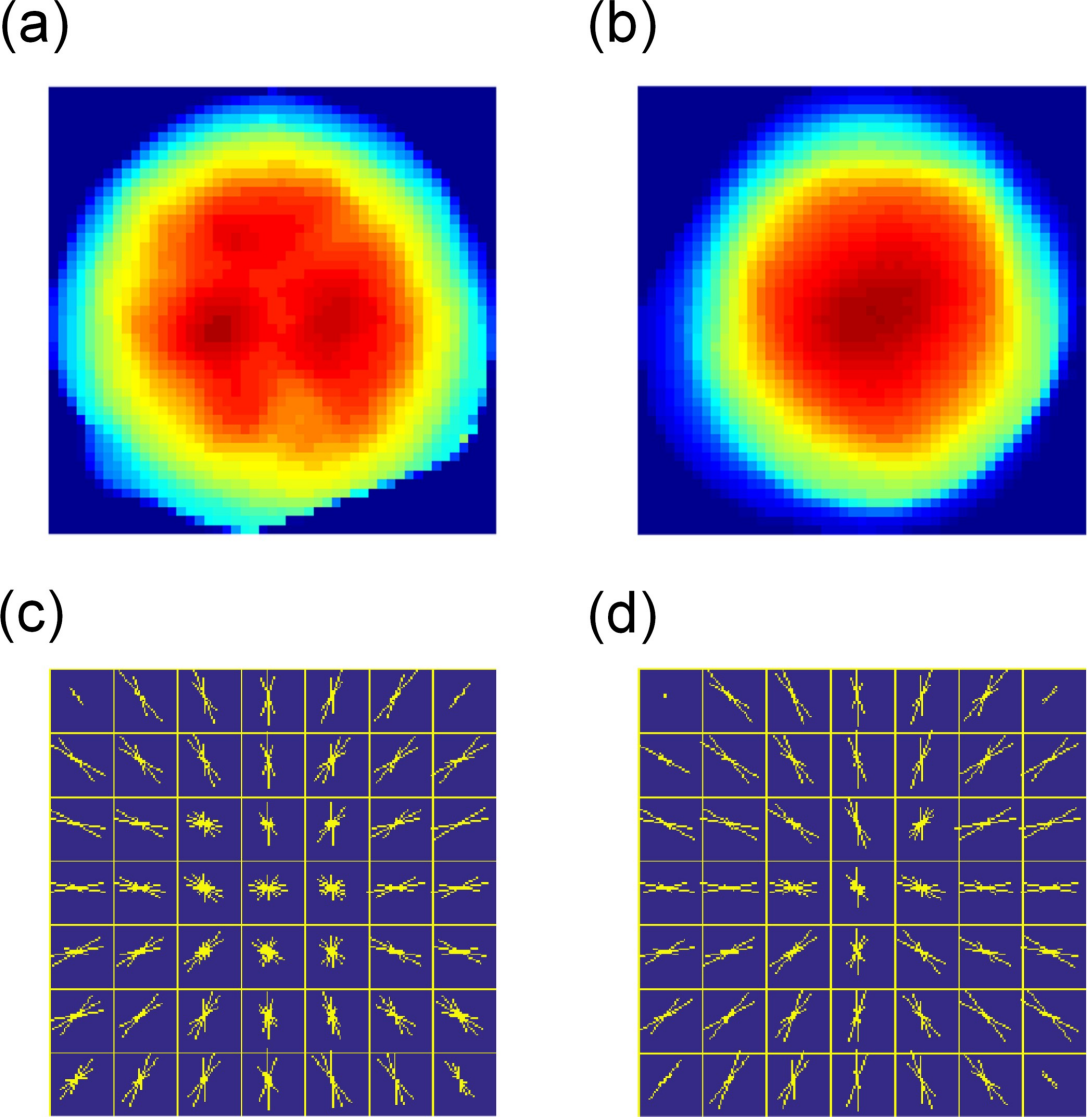
Representative QPM images and their visualized HOG features. (a) QPM image of cell line. (b) QPM image of WBC. In (a) and (b), pseudo color represents OPL. OPL is not normalized by diameter or path length. (c) Visualized HOG feature of (a). (d) Visualized HOG feature of (b). In (c) and (d), angles and lengths of the yellow lines in each compartment respectively represent orientation and strength of spatial gradient in OPL of the image.

A single cell contains organelles, which have different refractive indices [40,41]. As a result, a QPM can image subcellular structure in OPLs [42,43] in the lateral direction due to the distribution of refractive indices. Besides, it is reported that the structure might be related to malignancy [44–47]. As shown in Fig 5(A) and 5(B), OPL distribution of a CL cell is heterogeneous, whereas that of a WBC is smooth. These appearances can be clues for classification of cells.

As statistical subcellular-structures, five statistical parameters of the OPL/PL or OPL/D of single cells, namely, mean, standard deviation, maximum, skewness, and kurtosis, were utilized for the training data sets. Ensemble means of the five statistical parameters of OPL/PL and OPL/D over all 250 WBCs or 250 CLs are listed in Tables 1 and 2. After the above normalizations, the SVM classifier was trained on the five statistical parameters. The cost parameter (C) was searched for the maximum AUC. AUCs for each C, which varied from 2^−1^ to 2^8^, were calculated by means of five-fold cross validation.

**Table 1:** Ensemble mean values of statistical parameters of OPL/PL over all WBCs and CLs.

	Mean	Std. dev.	Max.	Skewness	Kurtosis
WBCs	0.0185	0.00877	0.0378	1.039	0.0124
Cell lines	0.0208	0.00659	0.0397	-2.073	4.95

**Table 2:** Ensemble mean values of statistical parameters of OPL/D over all WBCs and CLs.

	Mean	Std. dev.	Max.	Skewness	Kurtosis
WBCs	0.0146	0.00751	0.0267	0.334	1.235
Cell lines	0.0154	0.00609	0.0265	0.317	-0.9312

### Subcellular features of single cells by HOG descriptor

We propose extracting subcellular feature from QPM images of single cells by a HOG descriptor, and training SVM classifier on the features. The procedure of the feature extraction was shown in Fig 4. After the longitudinal and lateral normalization, HOG feature was extracted and the gradient was compensated for the lateral normalization.

A HOG descriptor (see S3 Fig) enhances the feature of an image because the HOG descriptor counts occurrences of certain gradient orientations in localized portions of an image. Actually, the HOG descriptor extracted such gradients in Fig 5(C) and 5(D). In these figures show the visualized features extracted by the HOG descriptor (simply referred to as “HOG features”) [48] of (a) the CL and (b) the WBC, respectively. An image of a single cell is divided into seven by seven compartments. The angles and lengths of the yellow lines in each compartment represent orientation and strength of spatial gradient in OPL of an image, respectively. In the preprocessing of the HOG feature extraction, images were normalized not only in the longitudinal but also in the lateral direction. After the longitudinal normalization in the same way as described in the previous section, Fig 4(B), the segmented images were resized to the size of 49×49-pixel images in Fig 4(C) because the HOG descriptor accepts only same-size images (i.e., the HOG descriptor is size-variant). The resizing of an image enhances or degrades the gradients. For instance, the gradient depicted by a red arrow is enhanced by the resizing shown in Fig 4(C). To compensate the enhanced gradients, in the right panel in Fig 4(C), OPL was divided by size-normalization factor *m*.

Training-data sets consisting of HOGs of 250 WBCs and HOGs of 250 CLs of QPM images normalized by path length or diameter were used. Maximum AUC was then found for by varying the cost of SVM parameters from 2^−1^ to 2^8^. AUCs for each parameter C were calculated by five-fold cross validation.

### Image acquisition and data processing

The proposed QPM is a kind of interference microscopy, so the interferograms were captured by a CCD device and send to a computer, they were processed on MATLAB (Mathworks, Inc.) software to retrieve phase delay caused by OPL of a cell, and the phase was unwrapped by Goldstein’s algorithm [49]. The QPM image of the cell is segmented so that it fit the size (width *w* and height *h*) in ImageJ. The segmented image (*w* by *h*) is divided by its path length or its diameter, and resized (size-normalization) to 49-by-49 pixels by factors m_x_ = 49/*w* and m_y_ = 49/*h*. Factor *m* is averaged value of *m*_*x*_ and *m*_*y*_. Enhancement of the spatial gradients of OPL due to the size-normalization was compensated by multiplying *m* to OPL in the resized image shown in Fig 4(C). After that compensation, HOG features of the resized-and-compensated image were extracted by utilizing the HOGDescriptor function in the open-source computer-vision library OpenCV (version 2.4.10). This function can setup the parameters of HOG, such as image size (49 by 49), block size (14 by 14), stride size (7 by 7), cell size (7 by 7), and number of orientation bins (9). These parameters of HOG were set to the values in parentheses. The part of the source code for utilization of OpenCV is shown in S1 Text. The HOG descriptor translated a two-dimensional image (49×49 = 2401 pixels) to vector ***x*** consisting of 1296 elements.

For the training data set used in machine learning, 250 HOG features (vectors) of WBCs (positive) and CLs (negative) images were collected. Since the sample size (250 pairs) was less than the number of elements (1296), a SVM with a linear kernel was used as the machine-learning algorithm. The SVM plots given vectors (*x*_*i*_) of two classes in high-dimensional space and finds the optimal hyper plane that maximizes the margin between the two classes. It then builds a SVM classifier trained on the training images. The decision (discriminant) function [30,50] is given as

$$d_{i}=\frac{w^{T}x_{i}+b}{\left\|w\right\|},$$ (3)

where ***x***_i_ is a vector of HOG, ***w*** is a weight vector, and *b* is a bias. If decision value *d* is positive, the cell having HOG ***x***_i_ is classified as a positive image (i.e., a WBC). An SVM library, LIBSVM[51] version 3.20, on MATLAB software was utilized for that purpose. In the option of function svmtrain, svm_type was set to C-SVC (-s 0), kernel_type was set to linear function (-t 0), and other options were set to default values. Five-fold cross validation was taken to find the maximum area under the ROC curve (AUC) by varying cost (-c xx) parameters of the SVM. Cost “C” is the parameter for the soft-margin cost function, which controls the influence of each individual support vector [50]. Function svmpredict predicts a decision value of a given HOG feature. The script of MATLAB is shown in S1 Text.

### Collection and processing of blood samples

**Fig 6:**
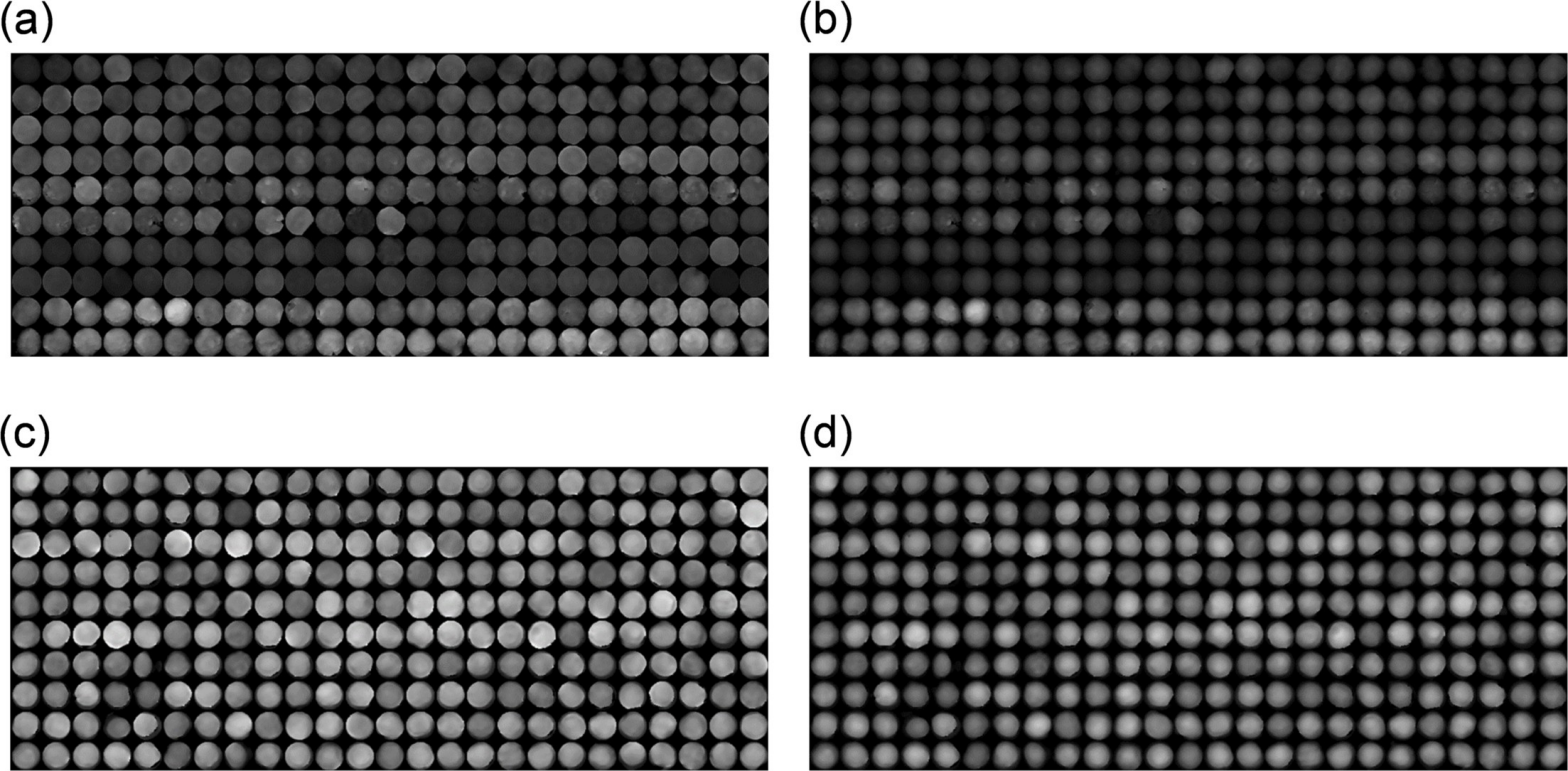
All QPM images (OPL) normalized by path-length or diameters. (a) OPL/PL: QPM images normalized by path lengths of cell lines; (b) OPL/D: QPM images normalized by diameters of cell lines; (c) OPL/PL: QPM images normalized by path lengths of WBCs; and (d) OPL/D: QPM images normalized by diameters of WBCs.

Blood samples were obtained from a healthy donor, and ethical approval for the study was obtained from the Institutional Review Board of Hamamatsu University School of Medicine (No.16-101), and the methods were carried out in accordance with the approved guidelines. Informed consent was obtained in a written form from each donor before sample collection. The subjects consented to cooperate after they were informed that they would not incur any disadvantage, that they could resign from the study, that the researchers were obliged to protect their privileged information, and that their identities would not be revealed. They were collected in tubes containing an anticoagulant (EDTA) and diluted with an equal volume of PBS. After carefully layering 4 ml of the diluted blood over 3 ml of Lymphoprep in a 15-ml centrifuge tube, the cells were centrifuged at 15,000 rpm at 4°C for 30 minutes. Mononuclear cells were collected from the sample/medium interface using a Pasteur pipette. The cells were diluted with PBS to reduce the density of the solution, pelleted at 1500×g for 10 minutes, and then incubated in VersaLyse Lysing Solution (1 ml/5×10^5^ leucocytes) for 10 minutes. They were pelleted again at 1500×g for 10 minutes to remove contaminating red blood cells and platelets. The obtained cells were suspended in PBS at 1.5×10^5^ cells/ml. All the QPM images taken of both WBCs and CLs are shown in Fig 6.

### Cells and cell culture

Human-colorectal-tumor cell lines HCT116, SW480, and DLD-1, human-pancreatic-tumor cell line Panc-1, and human-hepatocellular-liver-tumor cell line HepG2 were purchased from the European Collection of Authenticated Cell Cultures (ECACC, England, UK) and maintained in Dulbecco’s modified Eagle’s medium (DMEM) supplemented with 10% fetal bovine serum (FBS) at 37°C in a humidified atmosphere containing 5% CO_2_.

## Results

### OPL normalizations for size-invariant feature extraction

**Fig 7:**
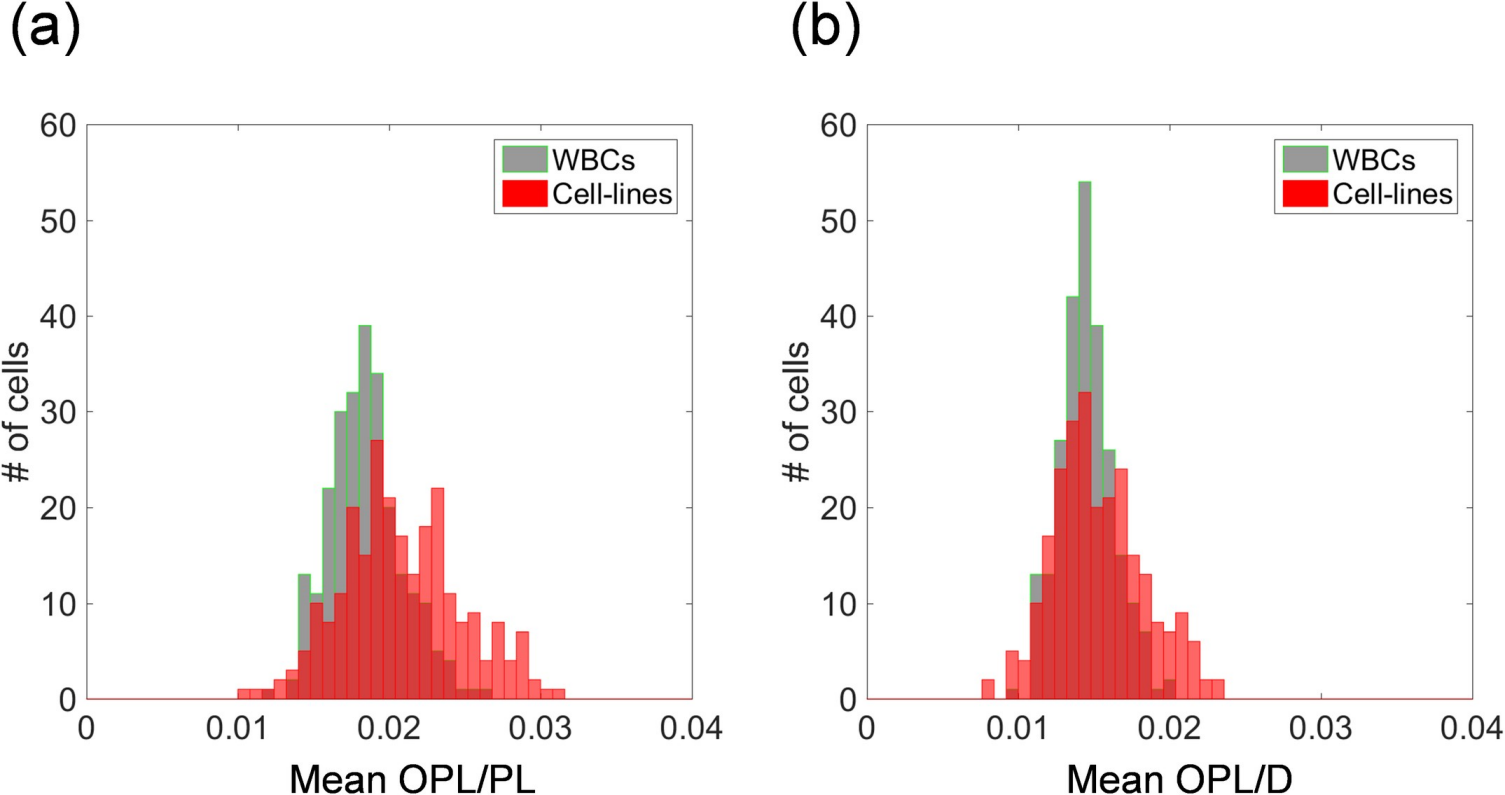
Statistical characteristics of two classes of training-data sets after two OPL normalizations. (a) Histograms of mean OPL normalized by path length (OPL/PL) over all WBCs and CLs and (b) histograms of mean OPL normalized by diameter (OPL/D) over all WBCs and CLs.

As for size-invariant feature extraction, OPL was normalized by path length or diameter. Histograms of mean OPL/PL or OPL/D over all 250 WBCs or 250 CLs of the training data sets are shown in Fig 7(A) and 7(B). Although the diameters of the two classes differ significantly (Fig 3(C)), the histograms of OPL normalized by PL or D in Fig 7 almost overlap.

### Training on statistical subcellular structures

**Fig 8:**
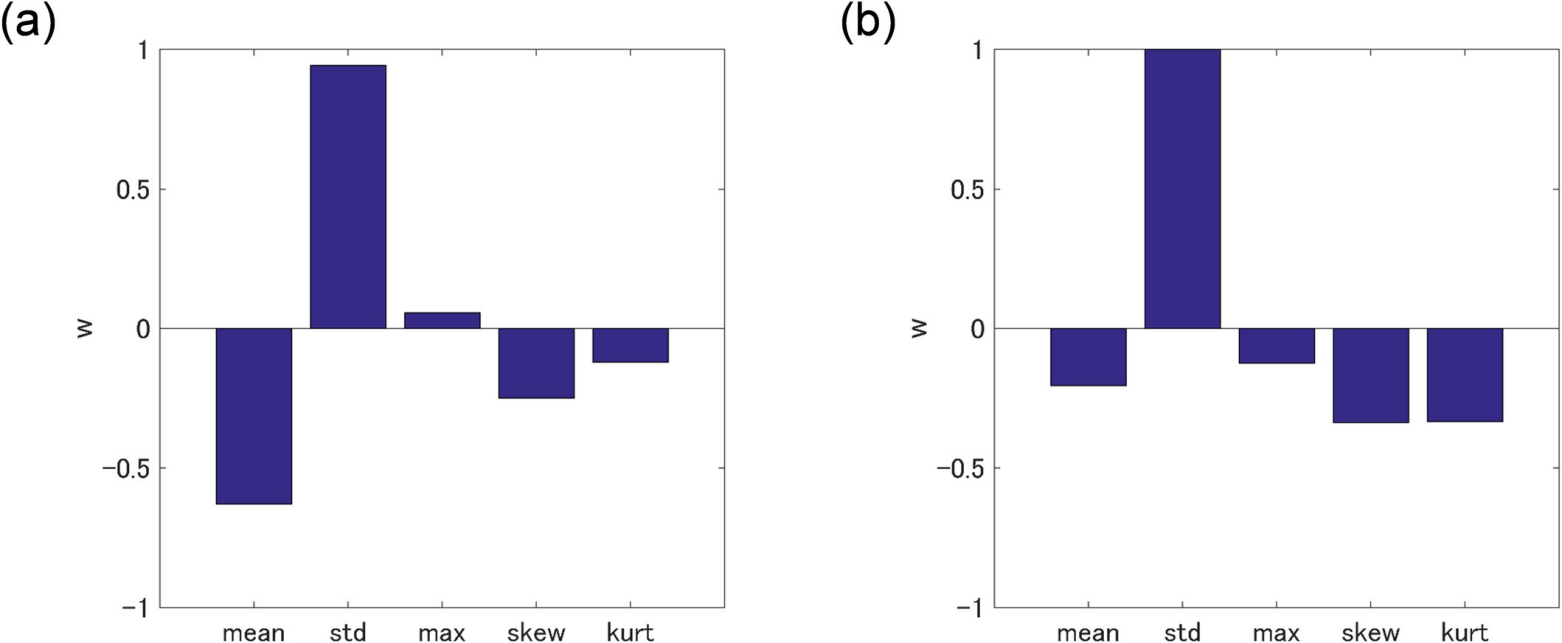
Weight vectors (*w*) of SMV classifiers visualized in five statistical parameters. (a) Weight vector for statistical parameters of OPL/PL and (b) weight vector for statistical parameters of OPL/D.

**Fig 9:**
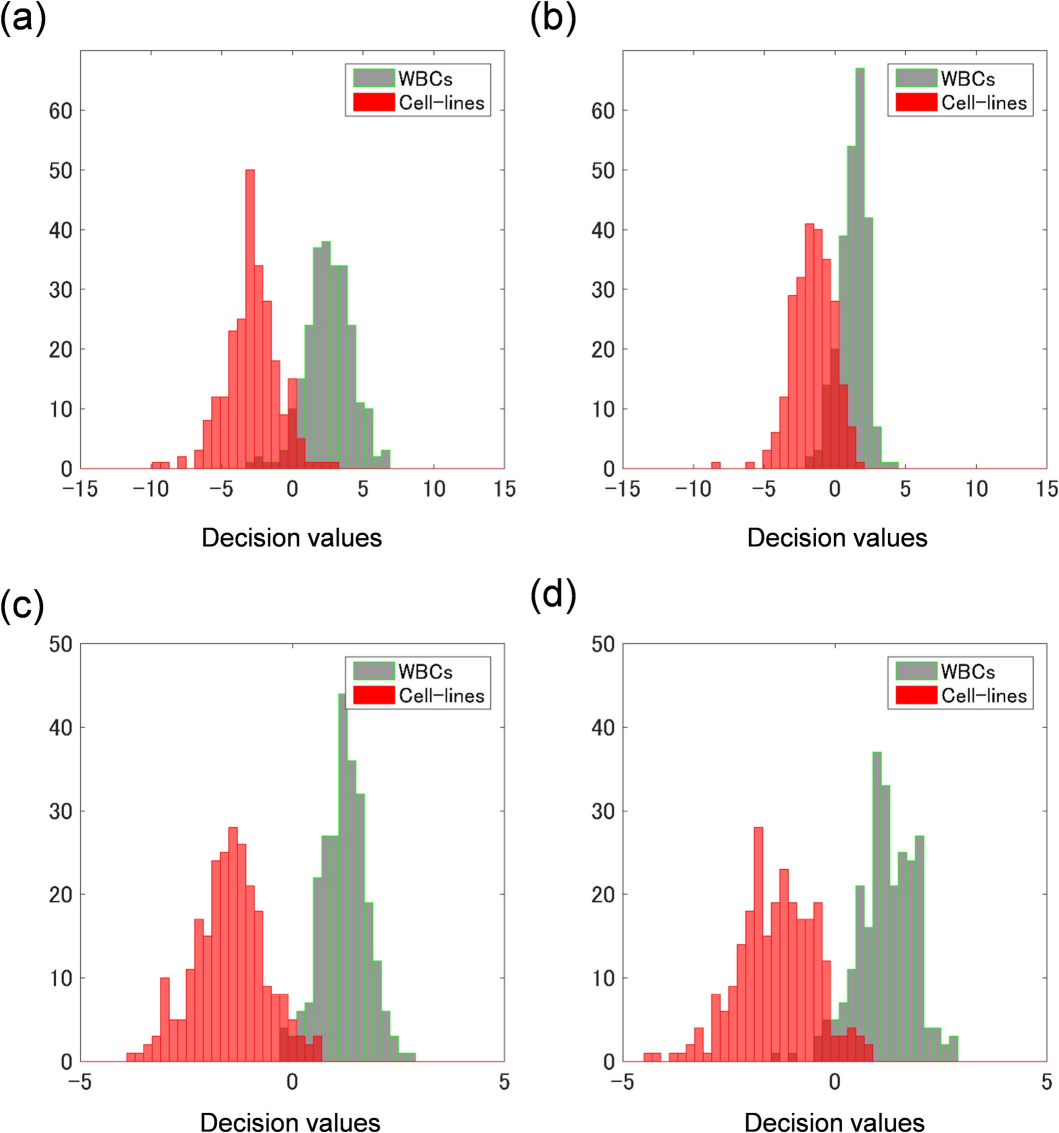
Histograms of decision values of training-data sets. Positive decision values for each cell are classified as a WBC, whereas negative decision values are classified as a cell line. Most WBCs (green bars) are classified as a positive decision value, and most cell lines (red bars) are classified as a negative one. The training data set consists of (a) statistical features of OPL/PL, (b) statistical features of OPL/D, (c) HOG features of OPL/PL, and (d) HOG features of OPL/D.

**Fig 10:**
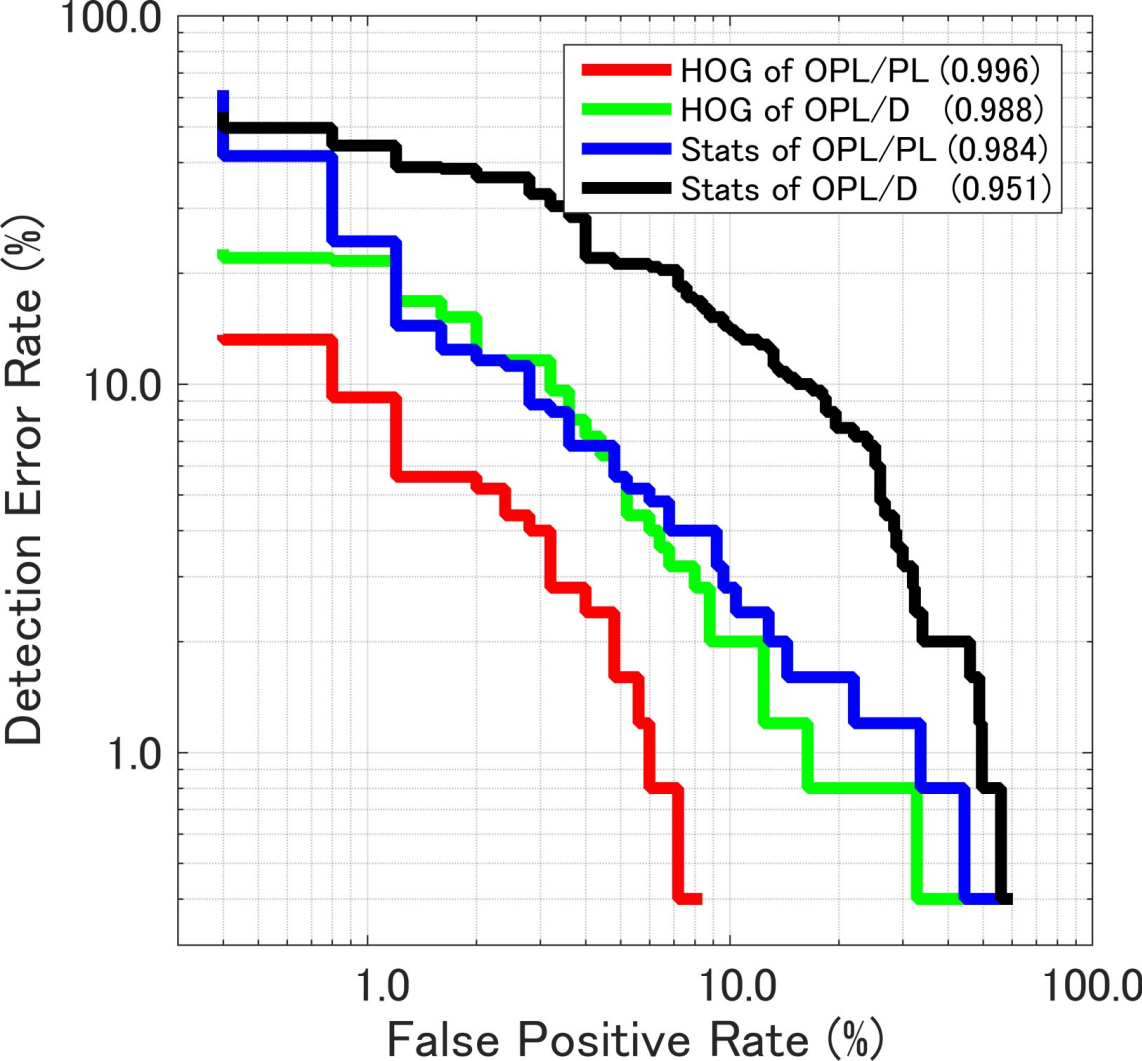
Detection-error trade-off (DET) curves for various training-data sets. Values in parentheses in the legend represent AUCs of their ROC curves.

The SVM classifier were built with the cost parameters C that gave the maximum AUC. Weight vectors [50] (***w***) of the classifiers are given in Fig 8. Both graphs indicate that the standard deviation is the main component for the classification in the case of both training data sets (OPL/PL and OPL/D). This result agrees with the fact that the standard deviations for the two classes (WBCs and CLs) significantly differ in Tables 1, 2, and S4 Fig. To evaluate the SVM classifier, histograms of decision values (i.e., outputs of the decision function given as ***x***_*i*_ in Eq 3) of WBCs and CLs of the training-data sets are shown in Fig 9. In Fig 9(A) and 9(B), most cells are properly classified, but some cells still overlap. The plot of sensitivity versus 1 –specificity is a called “receiver operating characteristic (ROC)” curve, while the plot of 1 –sensitivity versus 1 –specificity is called a “detection-error trade-off (DET)” curve [33]. DET curves for SVM classifiers trained by various training-data sets are shown in Fig 10. AUCs of ROC curves corresponding to the DET curves are shown in the legend of Fig 10.

### Training on subcellular structure by HOG descriptor

**Fig 11:**
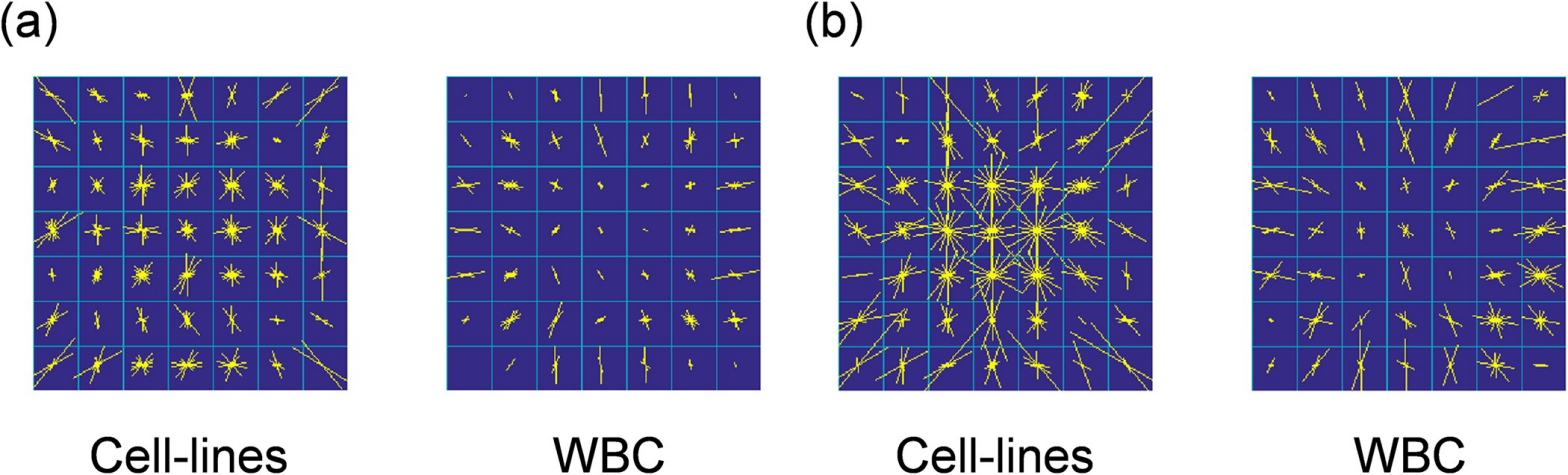
Weight vectors of SVM classifier found by parameter search visualized in seven-by-seven compartments. The angles and lengths of the yellow lines in each cell represent direction and strength of decline in an QPM image. (a) Weight vectors of QPM images (OPL) normalized by path length. Left side represents visualized weight vectors of HOG feature of cell lines. Right side represents vectors of WBCs. (b) Weight vectors of QPM images (OPL) normalized by diameter. In each panel, the left sides represent weight vectors of HOG feature of cell lines. The right sides represent the vectors of WBCs.

SVM Classifiers were built with the C parameters that gave the maximum AUC. Weight vectors (***w***) of the models are visualized in Fig 11, where the angles and lengths of the yellow lines in each cell represent orientation and strength of spatial gradient of OPL/PL or OPL/D in the images. Most arrows of the WBC in the center region are short, implying that the OPL/PL or OPL/D is uniform in that region. In contrast, the magnitude and orientation of the arrows of the HOG feature of CLs vary, especially in the center of the cell. These observations agree with the diagnostic criteria of cancer cells or abnormal cells.

To evaluate the SVM classifier histograms of decision values (output of decision function ***x***_*i*_ given in Eq 3) of WBCs and CLs of training-data sets are shown in Fig 9(C) and 9(D), respectively. The overlapping area of the histograms trained on HOGs of OPL/PL in Fig 9(C) is the smallest among all the panels in Fig 9. This result agrees with the facts that the corresponding DET curve is the lowest (red line) among the four DET curves and that the corresponding AUC (0.996) is the maximum listed in the legend in Fig 10.

### Interpretation of mechanism of classifications of cells

**Fig 12:**
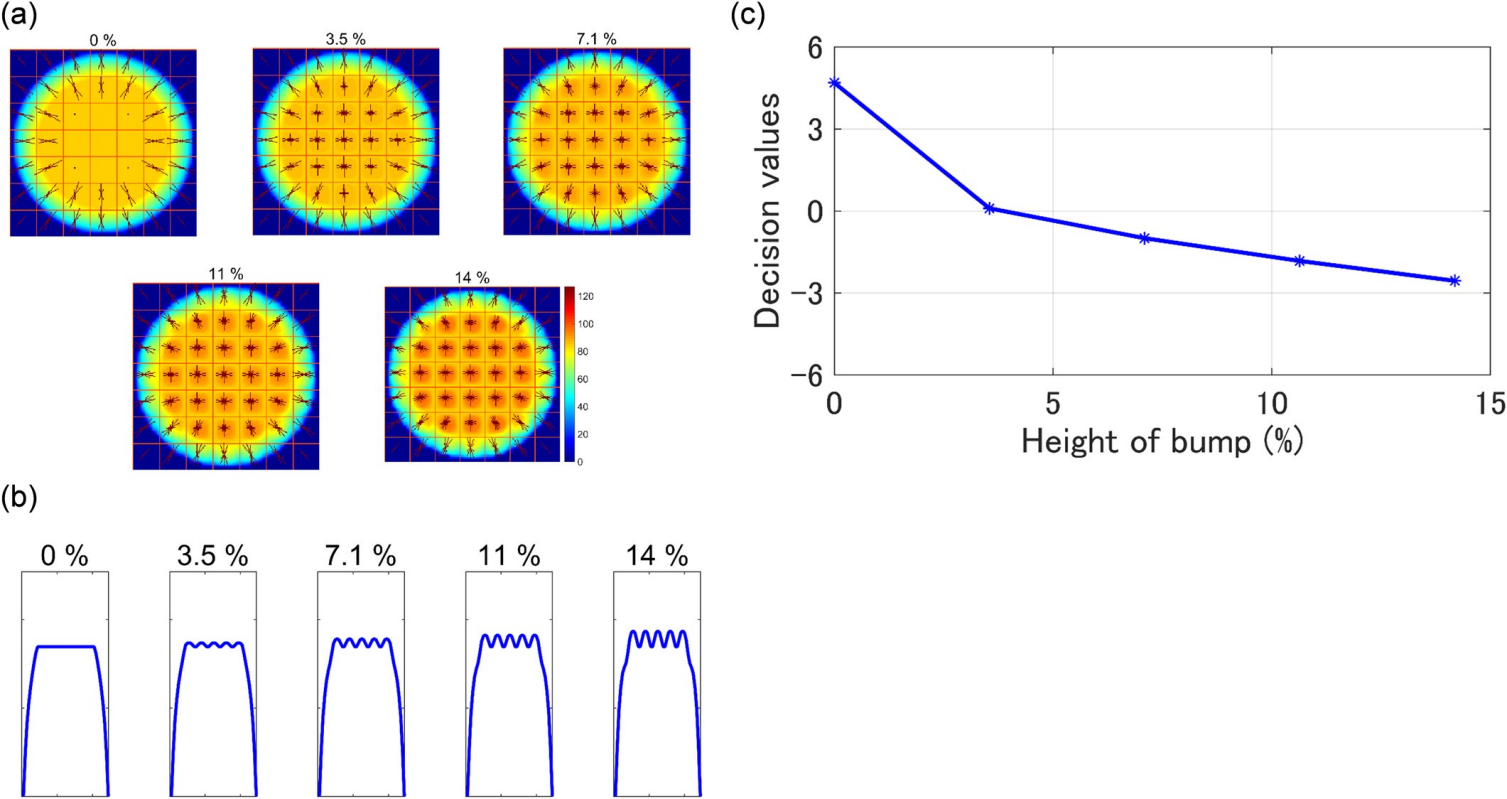
Classifications of heterogeneous phantoms. (a) Visualized HOG features superimposed on OPL/PL represented in pseudo color. The numbers above the figures are bump heights (%). (b) Cross sections of the phantoms. (c) Decision values of simulated phantoms with respect to bump heights are determined by the SVM classifier trained on HOG features of OPL/PL.

To understand what the SVM classifier “sees” in the images, phantoms with bumps on a flat surface were tested. Those bumps imitate organelle inside a cell. The five type of phantoms used are shown in Fig 12. Forty-nine (seven by seven) bumps are set on the flat surface. The heights of the bumps are variable from 0% to 14% with respect to the height of the flat surface. The visualized HOG features of three phantoms (7.1%, 11%, and 14%) shown in Fig 12(A) are similar to those of CLs shown in Fig 5(C). In Fig 12(A), the decision values of each phantom decease gradually from 4.7 to -2.6 with respect to the height of the bump on the top-hat phantom. The phantom with bump height of more than 3.5% is classified as CLs. These simulations suggest that cancer cells can be distinguished according to their intracellular heterogeneity from WBCs. These simulations suggest that the proposed classifier can recognize subcellular heterogeneity, especially in the center of the cell.

## Discussion

A method for label-free cell classification by computer-vision technologies for pattern recognition based on subcellular structures of QPM images of cells was demonstrated. This cell-recognition method differs from conventional methods in several respects. First, because QPM does not require a contrast agent nor staining to observe live cells, image acquisition by QPM is much less harmful to cells. This aspect is a great advantage for cell sorting based on features in QPM images. For example, the sorted cells can be cultivated to perform biological investigations. Second, cell classification is based on the heterogeneity of subcellular structures rather than the cellular outline used in conventional methods. The difference between weight vectors for WBCs and cancer cell lines in Fig 11 reflects the heterogeneity of subcellular components because compared with benign cells, malignant cells express a heterogeneous distribution of subcellular components [52]. As far as cancer CLs are concerned, the proposed classifier could find the difference between them.

In the sample preparation, Lymphoprep was used to separate mononuclear cells and platelets from red blood cells and granulocytes. Consequently, the target cells are lymphocytes, monocytes, and platelets. Platelets are smaller than the other cells. In morphology, nuclei of lymphocytes are round, and nuclei of monocytes are segmented. S3 Fig shows the projection of the RI along the optical axis (i.e., OPL) of a monocyte and lymphocyte. In contrast to these WBCs, nuclei of cultured cancer cells are expanded and bumpy. According to Figs 11 and 12, the proposed classifier appears to recognize the bumps which emulate cancer cell-lines’ bumps. Although a number of different types of WBCs exist, the SVM classifier successfully classified those different types of WBCs as one class distinguishable from another class of CLs (i.e., two-class classification).

In this paper, all the phase images were not refocused [53] after segmentations. As for all the experiments on imaging six kinds of cells (DLD-1, HCT116, HepG2, Panc-1, SW480, and WBC), only the one experimenter carefully focused cells after they are settled at the bottom of the sample chamber, by adjusting the position of the optical system including objective lenses so as to focus entire cells in the field of view. This adjustment gives rise to defocus for a larger or smaller cell than the cells having average diameters even though only one kind of cell is in the field of view. The distributions of predicted diameters from images of WBCs in Fig 3(C), and five types of CLs are shown individually in S5 Fig. The distributions of CLs are broader than those of WBCs, and their images were more blurred (smoother, not bumpy). This fact implies that defocusing mostly occurs with CLs, and increases their decision values (i.e. shift to positive). Consequently, the defocusing adversely affected the classification performances (especially, specificity and AUC).

A HOG descriptor is generally rotation-variant. It is used for detecting pedestrians on roads because they can be supposed to be stand-up (no rotation). In contract, a single cell has no orientation (i.e., no left and right and no upside down). We confirmed that the built SMV classifier is robust in relation to rotation of a cell in S6 Fig. We also confirmed that the sample size is sufficient by drawing a learning curve (see S7 Fig). These facts indicate that a HOG descriptor is applicable to feature extraction of a single cell.

Among the label-free, subcellular classifications, the most-reliable classifier is trained on subcellular structures (HOG features) of OPL/PL [Fig 6(A) and 6(C) and Fig 9(A) and 9(C)]. As shown in Fig 10, AUC of this model is 0.996. This value might result from the OPL normalization shown in Fig 4(B). OPL divided by PL [expressed by Eq (2)] enhances bumps on the line profile of OPL/PL in Figs 4(B) and 11. The randomly oriented yellow lines representing the visualized weight vector of cell lines in Fig 11(A) exit all compartments, whereas the randomly oriented yellow lines of cell lines in Fig 11(B) exit only the center compartments in three-by-three compartments.

In the statistical subcellular-structures classification, according to the box plot in S4 Fig, it is predicted that adding statistical subcellular parameters (standard deviation, skewness, and kurtosis) to the cellular outline (mean and maximum) improved classification (Fig 7, Fig 10) up to the level of HOG-based classification.

The sensitivity and specificity of the classification on the basis of cell size are the best among the other parameters in Table 3 because the sizes of the two populations we prepared in this research seldom overlap in Fig 3(C). In future applications of the proposed classification, we are focusing on CTC [54] detection in an enormous number of nucleated cells such as WBCs in blood. In this application, it would be difficult to classify CTCs on the basis of cell size because it has been reported that there is considerable overlap between the two cell populations [55]. It is reported that the morphology of some CTCs has been reported to resemble that of primary cancer cells [55]. This fact imply that we can use training images of cancer CLs substitute for that of CTCs.

**Table 3:** Performance of the classifications on the basis of various parameters.

	Stats of OPL/PL	Stats of OPL/D	HOG of OPL/PL	HOG of OPL/D	Diameter
Sensitivity [%]	95.2	87.6	98.4	95.2	100.0
Specificity [%]	93.2	86.8	95.2	94.8	99.2
Accuracy [%]	94.2	87.2	96.8	95.0	99.6
AUC	0.984	0.951	0.996	0.988	0.999

For future applications of this technology, we need to develop another technologies for acquiring QPM images of flowing cells, and sorting cells based on the label-free, subcellular classification This advanced technology will be a useful, non-cytotoxic, marker-free isolation method for CTCs. Since cell surface markers are dependent on tumor types and the tumor progresses, conventional surface-marker-dependent methods for detecting CTCs miss some populations of CTCs [56]. A cell-sorting system based on the label-free, subcellular classification can also be applied to classification of WBC examination. According to recent studies, tumor cells interfere with T-cells through co-inhibitory factors such as programmed cell death 1 and cytotoxic T-lymphocyte antigen 4, and they regulate the immune response, promoting tumor progression [57]. Antibodies blocking these immune checkpoint proteins have demonstrated clinical activity in the treatment of advanced malignant melanoma and non-small-cell lung cancer. In addition, inhibitory ligands and receptors that regulate T-cell effector functions in tissues are commonly overexpressed on tumor cells and non-transformed cells in the tumor microenvironment. These findings imply that tumor cells affect lymphocyte functions, which may result in a change of the morphological features of lymphocytes. Close examination of cancer patient-derived WBCs obtained by the proposed label-free, subcellular classification method may clarify the morphological features of tumor-educated WBCs, and it will be useful for estimating the effect of immune checkpoint inhibitors.

In summary, we developed a method for image classification of live cells. The classification was executed by computer-vision approach for pattern recognition of the subcellular structure of images. These images are label-freely acquired by QPM. This method, label-free subcellular classification, successfully differentiated human WBCs from cancer cell-line cells. We revealed that what features SVM sees in subcellular structure of a single cell and it provides a clue for classifying them label-freely. This method is expected to apply to detecting CTCs in a non-cytotoxic manner, thus providing good opportunities for studying intact CTCs.

## Supporting information

S1 Fig Spatial noise distribution of a background image. The standard deviation of the spatial background noise in a phase image was 5 nm (50 milli-radians) in single optical-path-length, while the mean maximum OPL of WBCs was about 300 nm. The noise distribution in S5 Fig has offset from the center. This may be due to not sufficient masking cells.
(TIF)

S2 Fig Images in the procedure for making a mask image, fitting a background image to a paraboloid, and subtracting the background image of a phase image from the original phase image. (a) original phase image with background curvature. (b) Paraboloid fitted to the phase image without masking cells. (c) phase image corrected by subtracting the paraboloid (b). (d) mask image to cover the cells on the phase image (a). (e) background image without cells (cells are masked by mask image (d). (f) paraboloid fitted to (a). (g) phase image corrected by subtracting the paraboloid (f); We compensated the difference in wave-fronts of the sample and reference light by fitting a background image to a paraboloid and subtracting it. In step one, a mask image (d) is extracted by fitting a paraboloid (b) to an original phase image (a) and setting a threshold (c) for distinguishing the background from objects. In step two, the original phase image is masked (e) by the mask image made in step one in order to obtain a background image without cells. Then, it was fitted to a paraboloid (f). Finally, a phase image corrected y subtracting the background image is obtained (g).
(TIF)

S3 Fig Projection images of cells in terms of OPLs and their gradients. Projection images of a cell in terms of optical path length (OPL) are shown in S1 Fig. OPL is proportional to refractive index (RI) or physical path length. HOG describes spatial gradients of OPL corresponding to the inclination of OPL in S1 Fig. The directions of the red arrows represent the directions of spatial gradients of OPL, and their lengths represent the magnitude of the spatial gradients. In practice, a captured QPM image is sectioned into 7×7 compartments (To avoid confusion, a cell, that is properly named in the field of computer vision, is referred to as a compartment), and the spatial gradient of OPL is visualized in each compartment. (a) schematic of a WBC, its profile of OPL, and visualized HOG feature (red arrows); and (b) schematic of a cancer cell, its profile of OPL, and visualized HOG feature (red arrows).
(TIF)

S4 Fig Characteristics of five statistical subcellular structures. Five statistical parameters are plotted in Box and whisker plots. The first quartile (Q_1_) and 3rd quartile (Q_3_) are boxed. Interquartile range is referred to as IQR. The upper whisker is Q3+1.5IQR, and the lower whisker is Q1-1.5IQR. Outliers are plotted as red crosses. Mean values are expressed as circles. The red boxes represent CLs, and the green boxes represent WBCs. (a) Five statistical parameters of OPL/PL and (b) five statistical parameters of OPL/D.
(TIF)

S5 Fig Distributions of predicted diameter of various types of cell-lines. Five types of cell-lines (DLD-1, HCT116, HepG2, Panc-1, and SW480) were imaged separately. We predicted the diameters of the segmented cells by averaging the width and the height of boundary box of a cell. No refocusing was done before segmentation of the cell in an image.
(TIF)

S6 Fig Robustness of HOG to rotation of cell images. The robustness of the SVM classifier trained on OPL/PL shown in Fig 9(C) against rotation of images was tested as follows. Two representative QPM images of phantoms were chosen: a heterogeneous hemi-ellipsoid phantom with a bump height of 11% for CLs (a), and a homogeneous hemi-ellipsoid with a top-hat phantom for WBCs (b). Two phantom models are shown in panel (a) and (b) respectively as maps of OPL/PL and their cross-sections. These phantoms were rotated from 0 to 350° in 10° steps and classified by the built classifier. In panel (c), the WBC phantom (green line) showed almost no change in the decision value with respect to rotational angles, and the CL phantom (red line) showed a slight fluctuation in the decision value (which remained in the minus range). These results suggest that the effects of rotation of an image or cell are relatively small and do not affect the classification.
(TIF)

S7 Fig Learning curve for sample sizes of HOG features of QPM images. It was confirmed that sample size is sufficient for a SVM by drawing the learning curve in S4 Fig. A SVM was trained on 250 images pairs (positive and negative image pairs). The images to be extracted HOG features are normalized by path length (OPL/PL). SVM parameter (C) is fixed at 16.
(TIF)

S1 Text Source codes for extracting HOG features, training and predicting them. (PDF)
