# Supplementary figures and images for: Label-free classification of cells based on supervised machine learning of subcellular structures
Source: PLoS One. 2019 Jan 29;14(1):e0211347. doi: 10.1371/journal.pone.0211347 (PMC6350988; doi:10.1371/journal.pone.0211347)

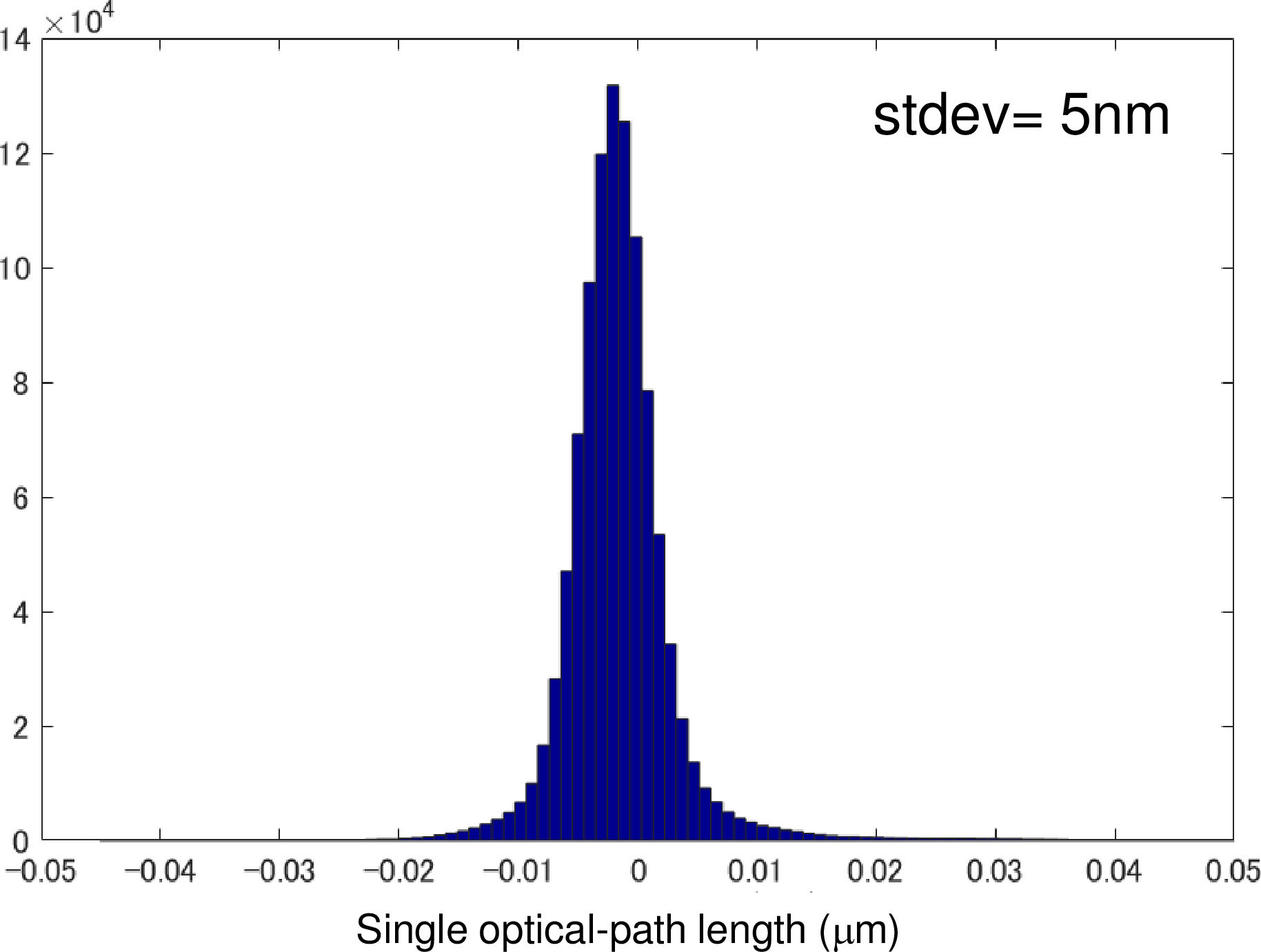

Supplement: S1 Fig — The standard deviation of the spatial background noise in a phase image was 5 nm (50 milli-radians) in single optical-path-length, while the mean maximum OPL of WBCs was about 300 nm. The noise distribution in S5 Fig has offset from the center. This may be due to not sufficient masking cells. (TIF) [file pone.0211347.s001.tif]

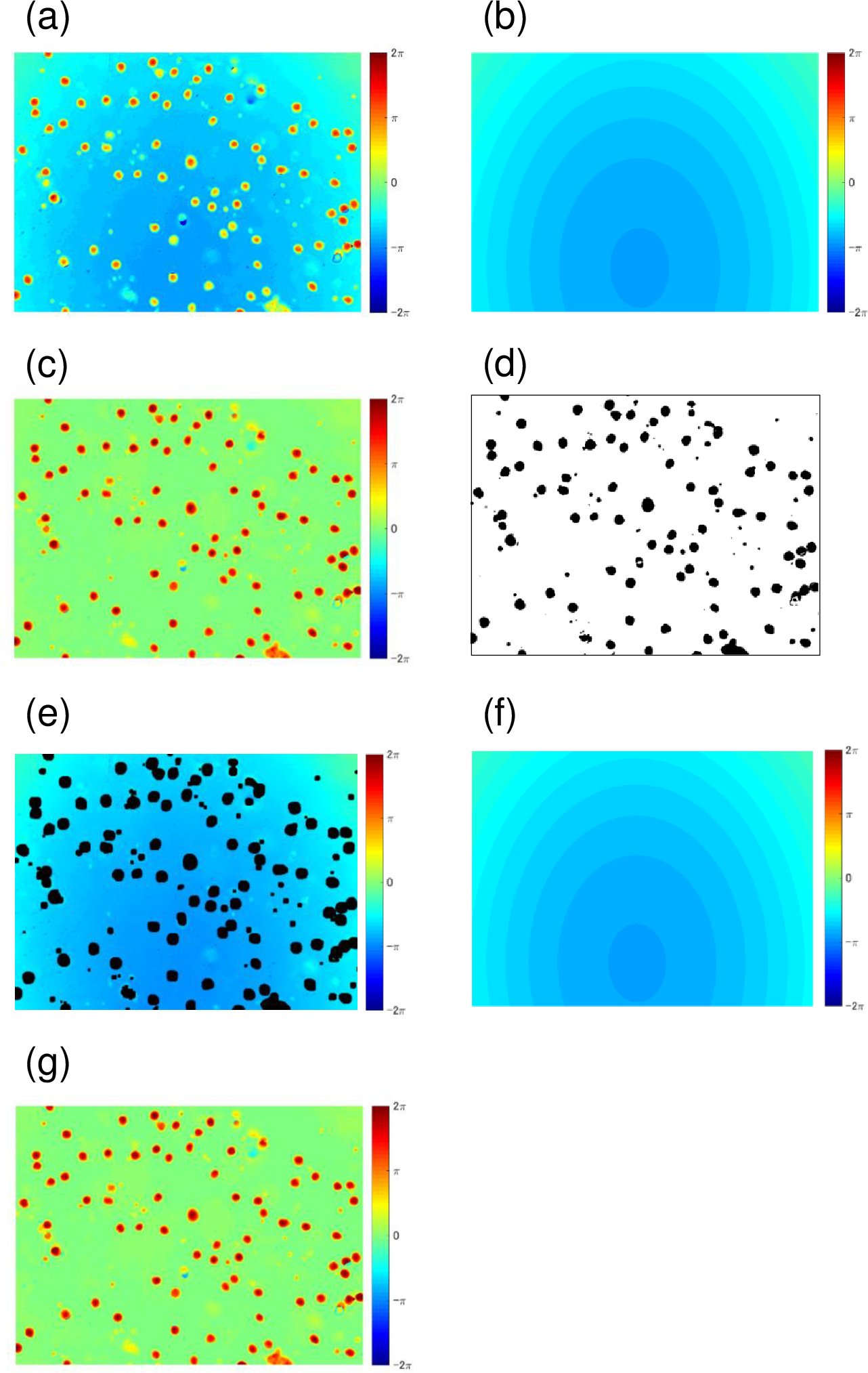

Supplement: S2 Fig — (a) original phase image with background curvature. (b) Paraboloid fitted to the phase image without masking cells. (c) phase image corrected by subtracting the paraboloid (b). (d) mask image to cover the cells on the phase image (a). (e) background image without cells (cells are masked by mask image (d). (f) paraboloid fitted to (a). (g) phase image corrected by subtracting the paraboloid (f); We compensated the difference in wave-fronts of the sample and reference light by fitting a background image to a paraboloid and subtracting it. In step one, a mask image (d) is extracted by fitting a paraboloid (b) to an original phase image (a) and setting a threshold (c) for distinguishing the background from objects. In step two, the original phase image is masked (e) by the mask image made in step one in order to obtain a background image without cells. Then, it was fitted to a paraboloid (f). Finally, a phase image corrected y subtracting the background image is obtained (g). (TIF) [file pone.0211347.s002.tif]

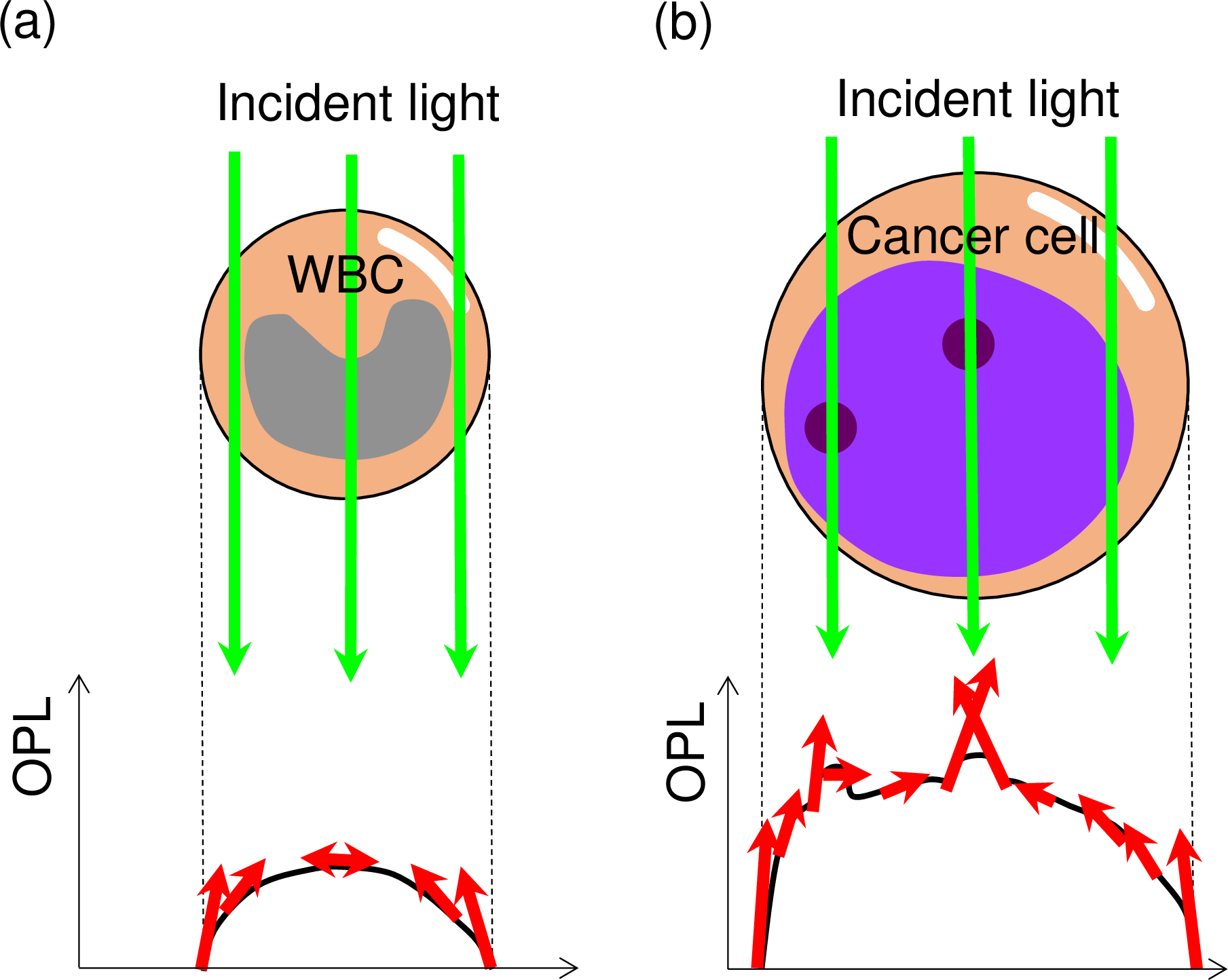

Supplement: S3 Fig — Projection images of a cell in terms of optical path length (OPL) are shown in S1 Fig. OPL is proportional to refractive index (RI) or physical path length. HOG describes spatial gradients of OPL corresponding to the inclination of OPL in S1 Fig. The directions of the red arrows represent the directions of spatial gradients of OPL, and their lengths represent the magnitude of the spatial gradients. In practice, a captured QPM image is sectioned into 7×7 compartments (To avoid confusion, a cell, that is properly named in the field of computer vision, is referred to as a compartment), and the spatial gradient of OPL is visualized in each compartment. (a) schematic of a WBC, its profile of OPL, and visualized HOG feature (red arrows); and (b) schematic of a cancer cell, its profile of OPL, and visualized HOG feature (red arrows). (TIF) [file pone.0211347.s003.tif]

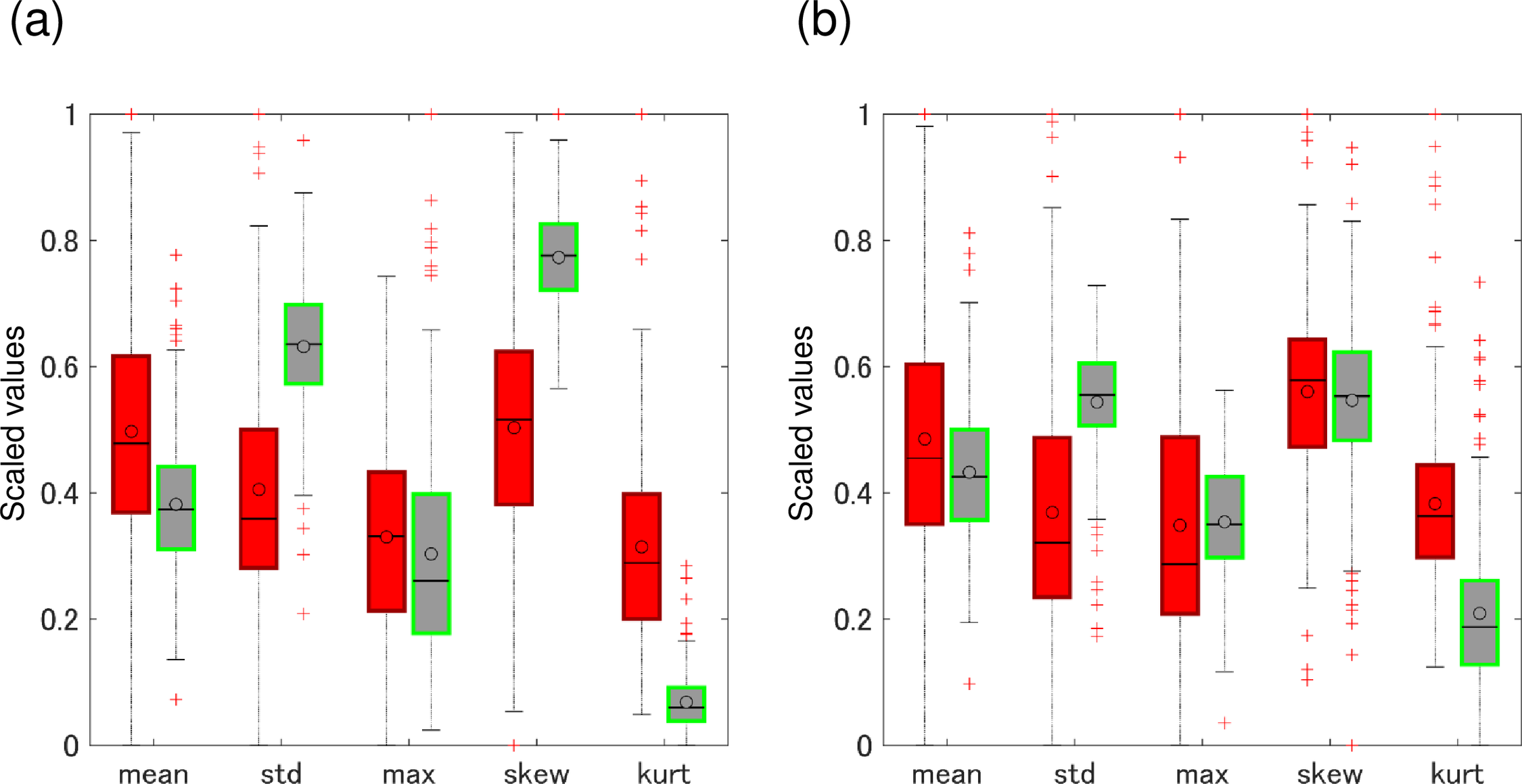

Supplement: S4 Fig — Five statistical parameters are plotted in Box and whisker plots. The first quartile (Q1) and 3rd quartile (Q3) are boxed. Interquartile range is referred to as IQR. The upper whisker is Q3+1.5IQR, and the lower whisker is Q1-1.5IQR. Outliers are plotted as red crosses. Mean values are expressed as circles. The red boxes represent CLs, and the green boxes represent WBCs. (a) Five statistical parameters of OPL/PL and (b) five statistical parameters of OPL/D. (TIF) [file pone.0211347.s004.tif]

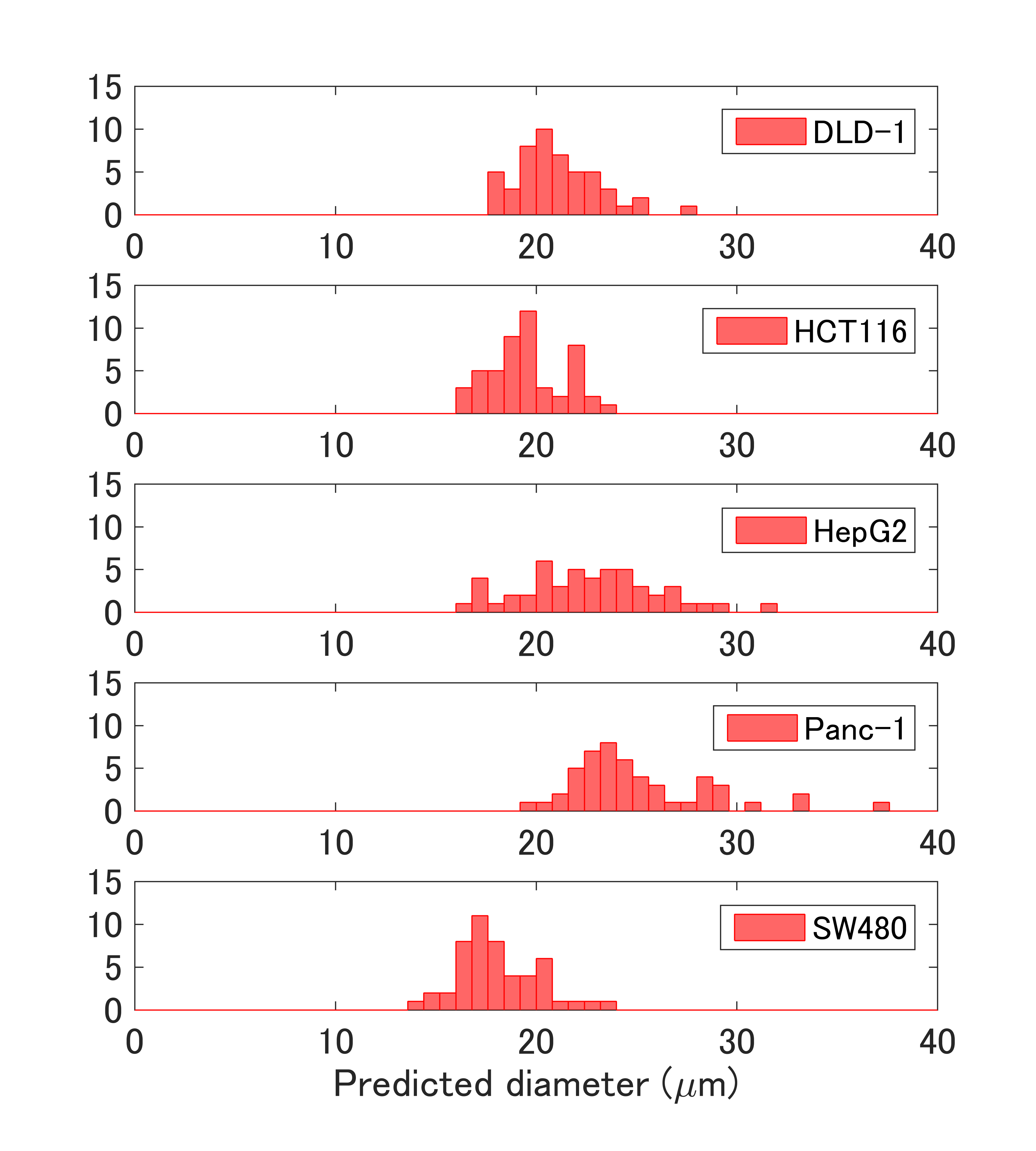

Supplement: S5 Fig — Five types of cell-lines (DLD-1, HCT116, HepG2, Panc-1, and SW480) were imaged separately. We predicted the diameters of the segmented cells by averaging the width and the height of boundary box of a cell. No refocusing was done before segmentation of the cell in an image. (TIF) [file pone.0211347.s005.tif]

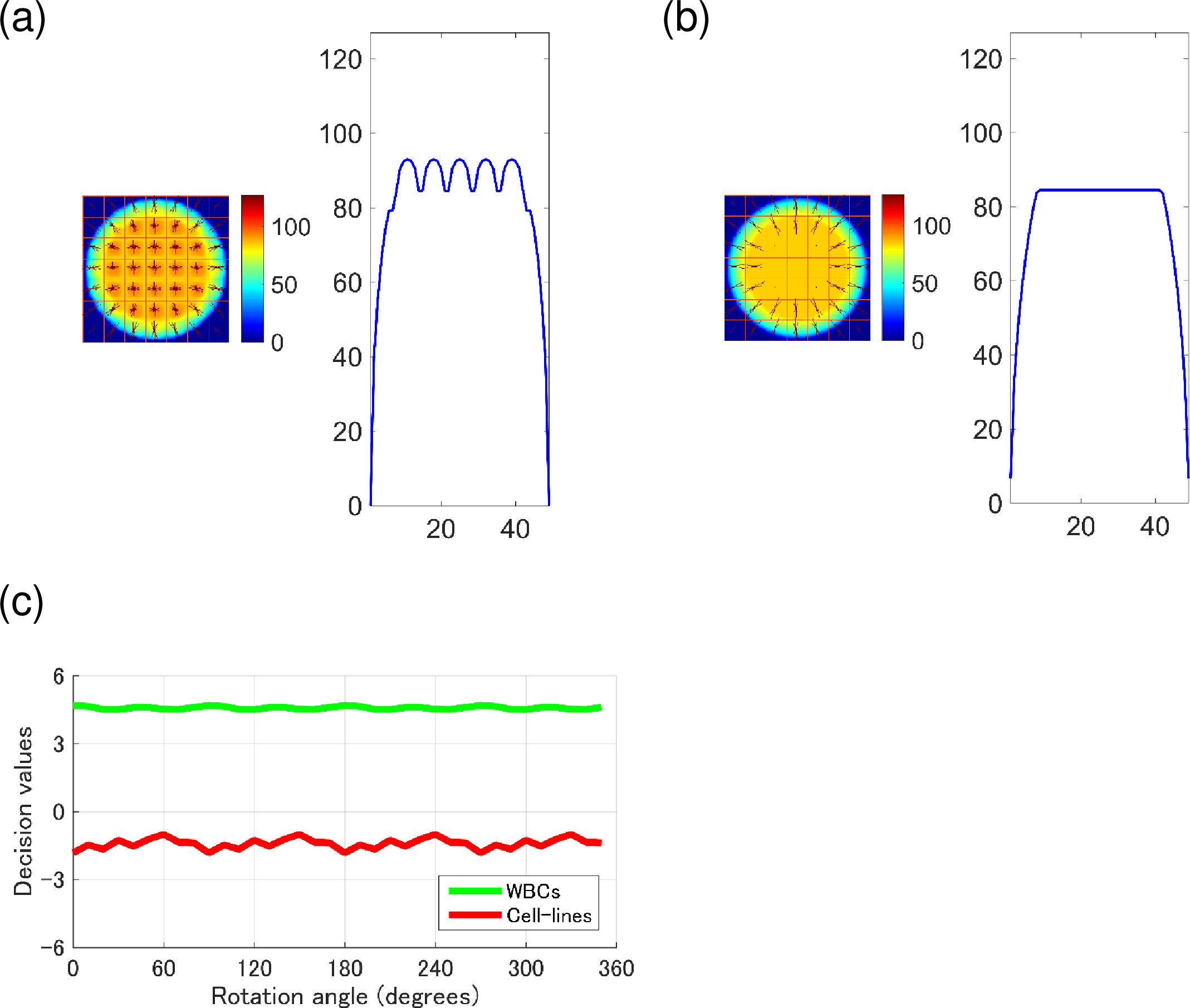

Supplement: S6 Fig — The robustness of the SVM classifier trained on OPL/PL shown in Fig 9(C) against rotation of images was tested as follows. Two representative QPM images of phantoms were chosen: a heterogeneous hemi-ellipsoid phantom with a bump height of 11% for CLs (a), and a homogeneous hemi-ellipsoid with a top-hat phantom for WBCs (b). Two phantom models are shown in panel (a) and (b) respectively as maps of OPL/PL and their cross-sections. These phantoms were rotated from 0 to 350° in 10° steps and classified by the built classifier. In panel (c), the WBC phantom (green line) showed almost no change in the decision value with respect to rotational angles, and the CL phantom (red line) showed a slight fluctuation in the decision value (which remained in the minus range). These results suggest that the effects of rotation of an image or cell are relatively small and do not affect the classification. (TIF) [file pone.0211347.s006.tif]

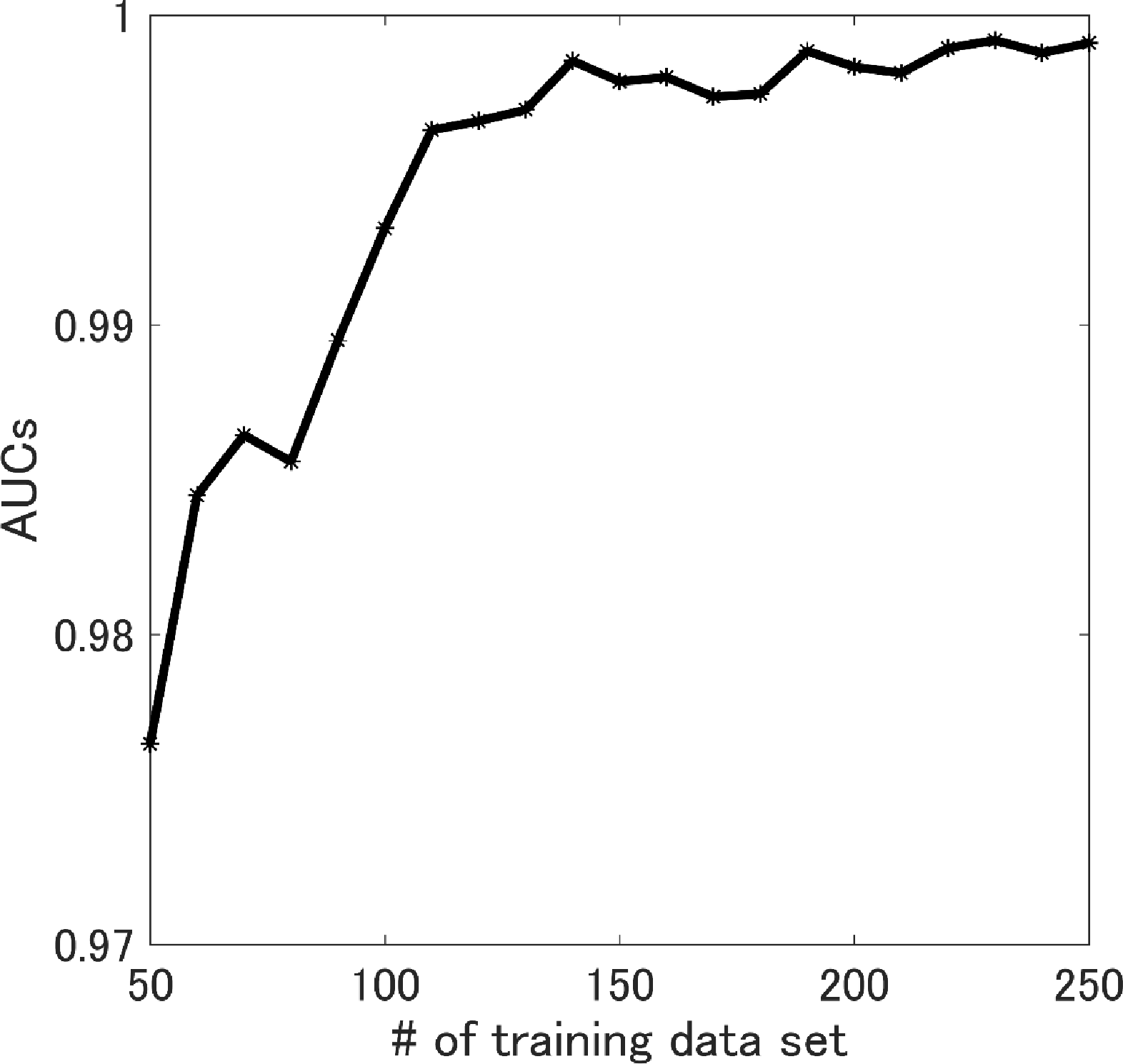

Supplement: S7 Fig — It was confirmed that sample size is sufficient for a SVM by drawing the learning curve in S4 Fig. A SVM was trained on 250 images pairs (positive and negative image pairs). The images to be extracted HOG features are normalized by path length (OPL/PL). SVM parameter (C) is fixed at 16. (TIF) [file pone.0211347.s007.tif]
