# Supplementary material for: Label-free classification of cells based on supervised machine learning of subcellular structures
Source: PLoS One. 2019 Jan 29;14(1):e0211347. doi: 10.1371/journal.pone.0211347 (PMC6350988; doi:10.1371/journal.pone.0211347)
Supplement: S1 Text — (PDF) [file pone.0211347.s008.pdf]

S4 Text. Source codes for extracting HOG features, training and predicting them.

A part of the source code for utilization of OpenCV for HOG extraction.

```
// C++ source code
using namespace cv;
…
cv::HOGDescriptor d( Size(49,49), Size(14, 14), Size(7, 7), Size(7, 7), 9 );
vector< float> descriptorsValues;
vector< Point> locations;
d.compute( img, descriptorsValues, Size(0,0), Size(0,0), locations );
…
```

A part of the script for utilization of MATLAB for training and predicting of SVM.

```
%% matlab script
…
libsvmwrite(fname, labels, sparse(features));
[label_vectors, instance_matrix] = read_sparse(fname)
…
model = svmtrain(training_label_vectors, training_instance_matrix,‘-s 0 -t 0 -c
xx);
…
[predicted_label, accuracy, decision_values] =
svmpredict(testing_label_vector, testing_instance_matrix, model);
…
```
